# Temporal variability in the growth-enhancing effects of different bacteria within the microbiome of the diatom *Actinocyclus* sp.

**DOI:** 10.3389/fmicb.2023.1230349

**Published:** 2023-08-07

**Authors:** Nine Le Reun, Anna Bramucci, Penelope Ajani, Abeeha Khalil, Jean-Baptiste Raina, Justin R. Seymour

**Affiliations:** ^1^Climate Change Cluster, University of Technology Sydney (UTS), Sydney, NSW, Australia; ^2^School of Life Sciences, University of Technology Sydney, Ultimo, NSW, Australia

**Keywords:** diatom-bacteria interaction, co-culture, microbiome, microalgae, *Actinocyclus*

## Abstract

Reciprocal metabolite exchanges between diatoms and bacteria can enhance the growth of both partners and therefore fundamentally influence aquatic ecosystem productivity. Here, we examined the growth-promoting capabilities of 15 different bacterial isolates from the bacterial community associated with the marine diatom *Actinocyclus* sp. and investigated the magnitude and timing of their effect on the growth of this diatom. In the presence of its microbiome, *Actinocyclus* sp. growth was significantly enhanced relative to axenic cultures. Co-culture with each of the 15 bacterial isolates examined here (seven Rhodobacteraceae, four Vibrionaceae, two Pseudoalteromonadaceae, one Oceanospirillaceae and one Alteromonadaceae) increased the growth of the diatom host, with four isolates inducing rates of growth that were similar to those delivered by the diatom’s full microbiome. However, the timing and duration of this effect differed between the different bacteria tested. Indeed, one Rhodobacteraceae and one Alteromonadaceae enhanced *Actinocyclus* sp. cell numbers between days 0–6 after co-incubation, five other Rhodobacteraceae promoted diatom cell numbers the most between days 8–12, whilst four Vibrionaceae, one Oceanospirillaceae and one Rhodobacteraceae enhanced *Actinocyclus* sp. cell abundance between days 14–16. These results are indicative of a succession of the growth-enhancing effects delivered by diverse bacteria throughout the *Actinocyclus* sp. life cycle, which will likely deliver sustained growth benefits to the diatom when its full microbiome is present.

## Introduction

Interactions between diatoms and bacteria shape microbial community dynamics and have important ecological implications as they influence both biological and chemical processes that structure the marine food web and govern biogeochemical cycling (Falkowski et al., 1998; Field et al., 1998; Liu et al., 2019; Le Reun et al., 2022). Diatoms exude dissolved organic carbon (DOC) that fuels the growth of heterotrophic bacteria, which subsequently remineralise these organic compounds and thereby potentially promote primary production (Azam and Malfatti, 2007; Falkowski et al., 2008; Worden et al., 2015). However, recent work has revealed that more complex interactions can occur between these marine microbes, with a diverse suite of sophisticated reciprocal chemical exchanges and signalling uncovered (Amin et al., 2015; Durham et al., 2015; Seymour et al., 2017; Cirri and Pohnert, 2019; Shibl et al., 2020; Barak-gavish et al., 2022). These interactions can involve the bacterial provision of various molecules, such as vitamins (i.e., B_12_, B_1_, B_3_) (Croft et al., 2005; Durham et al., 2015; Cooper et al., 2019), bio-available iron (Amin et al., 2009), growth-promoting hormones (Amin et al., 2015), antimicrobial compounds (Seyedsayamdost et al., 2011a; Bramucci et al., 2018), or other advantageous molecules (Sison-Mangus et al., 2014; Harvey et al., 2016).

Recent studies have also demonstrated that diatom-bacteria associations can be dynamic and very specific (Sapp et al., 2007; Needham and Fuhrman, 2016; Crenn et al., 2018). For example, *Phaeobacter gallaeciensis* can modify its behaviour to become an opportunistic pathogen depending on the growth stage of its algal host (Seyedsayamdost et al., 2011b). As the host diatom ages, it releases *p*-coumaric acid, which induces the production of algicidal compounds by the bacteria, rapidly killing the algae (Seyedsayamdost et al., 2011b). Several studies also highlighted that diatom-bacteria associations are highly specific, as bacteria foreign to a specific diatom are often detrimental or parasitic whilst being beneficial to their native host (Grossart et al., 2005; Sison-Mangus et al., 2014; Crenn et al., 2018; Stock et al., 2019).

In addition to the behavioural changes amongst phytoplankton-associated bacteria, bacterial community changes can occur over short time periods (Moejes et al., 2017). For instance, the microbiome of the diatom *Phaeodactylum* experienced a major shift in dominant bacterial taxa over 36 days, with a higher proportion of Rhodobacteraceae during its early growth stages, followed by an increase in Flavobacteriaceae and Pseudoalteromonadaceae (Moejes et al., 2017). These dynamic interactions have also been reported in the field during diatom blooms, with Rhodobacteraceae dominating during early phases of blooms, followed by Flavobacteriaceae dominating the later bloom phase (Luria et al., 2016; Needham and Fuhrman, 2016; Sison-Mangus et al., 2016). These studies highlight the complex ecological dynamics of diatom-bacterial interactions and suggest that temporal shifts in each partners metabolism and physiology may underpin highly complex associations.

Whilst laboratory studies have provided important insights into the chemical exchanges and metabolic dependencies between diatoms and bacteria, they have either examined the effect of the full microbial consortia on algal growth through a reseeding experimental design, or the effect of individual bacteria on phytoplankton in co-culture experiments (Cirri and Pohnert, 2019; Shibl et al., 2020). Additionally, few studies have examined the individual effects of more than three different bacterial isolates on the same algal species (Gärdes et al., 2012; Sison-Mangus et al., 2014; Behringer et al., 2018; Johansson et al., 2019). As a consequence, we still do not know what proportion of a diatom’s microbiome imparts benefits to its growth, and how the effect of these different bacteria changes over time.

Here we focussed on *Actinocyclus* sp., a globally distributed marine diatom that is abundant in planktonic communities (Malviya et al., 2016; Lafond et al., 2020). We single-cell isolated *Actinocyclus* sp. from an oceanic site near Sydney in Australia and examined how bacteria isolated from the same water influenced the diatom’s growth. We then examined shifts in these diatom-bacteria relationships over short time-periods.

## Methods

### Diatom isolation and culture conditions

Fresh diatom cultures were established from water samples (surface water plankton tow) collected from the Port Hacking oceanographic station, which is located on the south-eastern Australian coast near Sydney (34° 05.00 S, 151° 15.00 E). Port Hacking is a long-running time-series site within the Integrated Marine Observing System (IMOS) National Reference Station (NRS) network (Brown et al., 2018). Diatoms were isolated using drawn out Pasteur pipettes (micropipettes) under light microscopy (maximum magnification 200X). Extracted cells were initially grown in 24 well plates containing sterile seawater diluted 50:50 with f/2 medium (Andersen et al., 2005). Once cultures were established, they were transferred into 50 mL culture flasks (Falcon) and maintained in a constant temperature incubator at 21°C, with a light intensity of 60–70 μE m^−2^ s^−1^ and a 12:12 light/dark cycle. In addition to its global distribution in planktonic communities, we chose this diatom species due to its relatively large size (~50 μm) and because it was non-chain-forming, making it suitable for accurate flow cytometry enumeration.

### Diatom identification: 18S rRNA gene sequence and TEM microscopy

For taxonomic identification, diatom cells were grown for 7 days, centrifuged at 2000 g for 5 min. DNA was extracted from the resultant pellet using a DNeasy PowerWater Kit following the manufacturer’s instructions (Qiagen). The full 18S rRNA gene was then amplified using the 18SmoonF and 18SmoonR primer set (Supplementary Table S1), with the following PCR cycle conditions: 94°C for 10 min; 30 cycles of: 94°C for 1 min, 55°C for 2 min, and 72°C for 3 min; followed by a final extension at 72°C for 10 min and cooling at 4°C. PCR were performed using a Thermal Cycler (Veriti 96-well Fast thermal Cycler, Applied Biosystems) and the product sequenced using Sanger sequencing (Australian Genome Research Facility; AGRF). The resulting sequence was compared to the National Centre for Biotechnologies Information (NCBI) database using the online BLASTn tool.

Transmission Electron Microscopy (TEM) was also used to unequivocally identify the diatom to the genus level based on distinct morphological features (pseudonodulus, central annulus, labiate processes, and areolation pattern). For TEM examination, cultures were preserved in Lugol’s iodine prior to cleaning using the method of Hasle and Fryxell (1970). Once cleaned, 3 μL of culture were placed on formvar-coated copper grids and loaded into a FEI Tecnai T20 TEM (LaB6), operated at a high tension of 120 kV and equipped with a Gatan 894 CCD 2 k × 2 k camera. Frustule characteristics and morphometrics were quantified using Image J1^1^ and guided by Schmidt (1878) and Hasle and Syvertsen (1996).

### Bacterial isolation and identification

A total of ~150 bacteria strains were isolated from the microbiome of *Actinocyclus* sp. and other diatom strains (all isolated from the environment at the same time from the same plankton tow sample) 20 days after the establishment of the cultures (Supplementary Table S2). Each bacterial strain was isolated and grown to purity by repeated plating on 1 and 10% Marine Agar (Difco) incubated at 21°C in the dark.

For identification, each bacterial isolate was grown for 24 h in 100% Marine Broth (Difco Marine Broth 2,216) at 21°C, under 160 rpm shaking. DNA was then extracted and the 16S rRNA gene amplified by PCR using the 27F and 1492R primer set (~1,500 bp, Supplementary Table S1), which was performed using the following PCR cycle conditions: 95°C for 10 min; 30 cycles of: (i) 95°C for 30 s, (ii) 50°C for 30 s, and (iii) 72°C for 90 s; followed by a final extension at 72°C for 10 min. Samples were then Sanger sequenced (Australian Genomic Research Facility). Sequences were quality trimmed using Geneious software (v2022.1.1) and aligned using the MUSCLE alignment method. Bacteria were identified to the species level if their sequences shared >99% similarity with an entry in BLASTn database, and otherwise identified to the genus level. For comparison with the microbiome data, bacterial DNA was also sent to AGRF for Illumina sequencing on a MiSeq platform targeting the V1-V3 region of the 16S rRNA gene (~ 490 bp, Supplementary Table S1). Sequences were processed as per the microbiome analysis below with the BLAST tool used to match the bacterial isolates ASVs against the *Actinocyclus* microbiome with a > 97% percent identity threshold.

### Microbiome analysis

To characterise the bacterial component of the *Actinocyclus* sp. associated bacterial assemblages (free-living and attached bacteria), 16S rRNA amplicon sequencing was used. A 7-day old *Actinocyclus* sp. culture was inoculated at a starting concentration of 5,000 cells mL^−1^ and sampled at days 2, 4, 6, 8 and 12. At each time point, four replicates of 50 mL were filtered through 0.22 μm pore-size polycarbonate membrane filter (Millipore). DNA was subsequently extracted from filters using the Dneasy Power Water Kit (Qiagen) and quantified with a Nanodrop spectrophotometer (Thermo Scientific). Samples were then sequenced using the Illumina MiSeq platform targeting the V1-V3 region of the 16S rRNA gene (Supplementary Table S1). A negative control (DNA extraction with no sample) was also sequenced.

All sequences were processed through the DADA2 pipeline [https://github.com/martinostrowski/marinemicrobes/tree/master/dada2 (accessed on 31 May 2021)]. Briefly, the DADA2 R package was used to remove primers, filter and trim quality terminal error (16S rRNA trunc lengths R1 = 270, R2 = 250), denoise, merge, and remove chimeras from the dataset (Callahan et al., 2019). ASVs were assigned using a naïve Bayes classifier based on SILVA 138.1 and a bootstrap cut-off >50% (Wang et al., 2007; Yilmaz et al., 2014). ASV abundance tables were then normalised using the cumulative sum scaling approach implemented in the metagenomeSeq R package which account for uneven sequencing depths (Paulson et al., 2013).

### Axenic protocol

**Figure 1:**
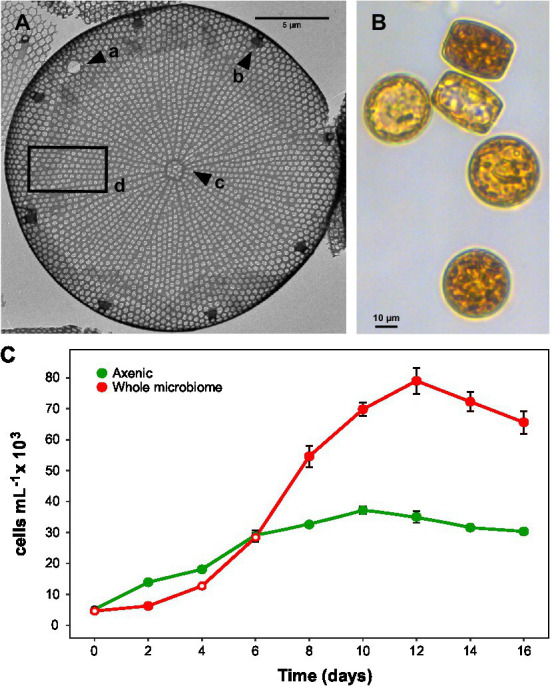
*Actinocyclus* sp. morphology and growth. **(A)**: Transmission electron microscopy image with a: large pseudonodulus close to the valve mantle, b: marginal ring of large labiate processes, c: central annulus irregular in shape and d: valve face with areolation radial and fasciculate and rows slightly curved. **(B)**: Light microscopy image (200X) of *Actinocyclus* sp. **(C)**: Growth curves of axenic and non-axenic (with their full microbiome) *Actinocyclus* sp. cultures, as measured by cells mL^−1^ counts performed using flow cytometry (gates based on chlorophyll-a). Filled circle refer to time points that were significantly different from the axenic controls (Simple Main Effect test, *p* < 0.05, Supplementary Table S4), whilst empty circles were not statistically different. Error bars represent the standard error of the mean (*n* = 4).

Axenic cultures of *Actinocyclus* sp. (Figure 1B) were generated (*n* = 3) using the protocol described by Shishlyannikov et al. (2011) with minor modifications. Approximately 50 mL of each mid-late exponential phase *Actinocyclus* sp. culture was gravity-filtered through a 5 μm pore-size polycarbonate membrane filter (Millipore). The filter was then transferred into sterile seawater and the cells were gently resuspended by inverting, before the addition of 20 μg mL^−1^ Triton X-100 (Sigma Aldrich). After 1 min of Triton X-100 exposure, the suspension was filtered through a new 5 μm filter and rinsed with sterile seawater before being transferred to fresh f/2 media containing a cocktail of antibiotics, including: ciprofloxacin (Sigma Aldrich; final concentration 20 μg mL^−1^), ampicillin (Sigma Aldrich; final concentration 100 μg mL^−1^), gentamycin (Sigma Aldrich; final concentration 67 μg mL^−1^) and streptomycin (Amresco; final concentration 50 μg mL^−1^). Cells were then incubated under regular growth conditions for 7 days. After that time, 1 mL of culture was transferred into fresh f/2 media. To verify that the cultures were axenic, we followed the procedure of Larsson et al. (2022), whereby treated cultures were considered axenic only after: (1) no bacterial growth was identified when 1 mL of algal culture was inoculated into 100% Marine Broth for 7 days; (2) drop-plating of algal culture onto 100% Marine agar plates resulted in no bacterial growth; and (3) no bacterial population was present in algal cultures stained with SYBR Green (Sigma-Aldrich S9430) and enumerated with flow cytometry. Finally, DNA from these cultures (*n* = 3) was also extracted using Dneasy Power Water Kit (Qiagen), the V1-V3 region of the 16S rRNA gene was amplified and sequenced on a MiSeq platform (2 × 300 bp) (Illumina) at AGRF. All reads were identified as chloroplast, confirming the *Actinocyclus* sp. cultures were indeed axenic.

To ensure that differences in growth observed between cultures of *Actinocyclus* with its whole microbiome and axenic cultures were not due to the stress induced by the axenic protocol (*cf.* Figure 1C), cultures were acclimated for ~12 transfers (i.e., ~6 months) before starting the co-culture experiments. Furthermore, we also performed a “reseeding” experiment, whereby we added the initial microbial consortia back into the axenic cultures (Supplementary Figure S1). After growing axenic cultures, and cultures with the whole microbiome side by side for 7 days, the cultures with the whole microbiome were pooled together and 100 mL filtered through a 5 μm pore-size polycarbonate membrane filter (Millipore); the filtrate, containing the bacteria, was collected in a sterile bottle for use in subsequent steps. The remaining cultures with the whole microbiome (not filtered) were enumerated using flow cytometry and diatom cells inoculated to 5,000 cells mL^−1^ into fresh f/2 media (*n* = 4). Filtrate was diluted to 1 × 10^6^ cells mL^−1^ in axenic cultures inoculated to 5,000 cells mL^−1^ into fresh f/2 media (‘reseeded’ diatom) to match the density of bacteria present in the culture with the whole microbiome at day 0 (*n* = 4). The remainder of axenic cultures were diluted into fresh media to 5,000 cells mL^−1^, which served as controls (*n* = 4). Each culture was subsampled every two days, whereby 200 μL aliquots were fixed with glutaraldehyde (2% final concentration, G6257 Sigma-Aldrich) and both diatom and bacterial cells were quantified using flow cytometry as described below. The axenic cultures were checked for contamination throughout the course of the study by (1) drop-plating on 100% Marine agar plates and (2) flow cytometry (following staining with SYBR green).

### Quantification of diatom and bacterial cells

A 200 μL aliquot was sampled every 2 days and cells were enumerated using a flow cytometer (CytoFLEX LX, Beckman Coulter). The samples were analysed at a flow rate of 30 μL min^−1^ for diatom and 25 μL min^−1^ for bacteria. For diatom enumeration, samples were fixed with glutaraldehyde (2% final concentration) and incubated at 4°C for ~20 min. For bacterial enumeration, samples were first fixed with glutaraldehyde and after 20 min stained with SYBR Green I at 4°C for 15 min in the dark (final concentration 1:10,000, Life Technologies). Fluorescence was detected with blue laser (488 nm) excitation and a combination of 690–50 and a 525–40 nm bandpass filter. Diatom populations were identified and quantified according to chlorophyll-*a* (B690-50-A) vs forward scatter area (FSC-A), and SYBR Green fluorescence vs side-scatter area (SSC-A) for bacteria (CytExpert v2.4 software). To ensure accuracy of the gating settings and volume of samples processed between sampling time-points, fluorescent QC beads were used (B53230, CytoFLEX Daily QC Fluorospheres) and the flow cytometer regularly calibrated following the manufacturer’s instruction.

### Co-culture establishment and bacterial isolates selection

Out of the 150 bacteria isolated, we pre-selected 30 candidates for further examination based on their taxonomy and their likelihood to interact with diatoms. We subsequently performed a pre-screening experiment of their effect on *Actinocyclus* growth using co-culture plate assays. Each of the 30 co-culture was established in 1 mL volume in 48 well-plates (for details on co-culture preparation refer to the paragraph below). Plates were incubated as described above and algal growth monitored every 2 days for 16 days. Algal growth was estimated based on chlorophyll-a fluorescence (455 nm excitation, 680 nm emission) measured on an infinite M1000 pro plates reader (Tecan). Fluorescence was acquired from the bottom of the plate with 16 reads per well, a flash frequency of 400 Hz, 20 μs integration time, no lag time and 10 ms settling time. This process allowed us to narrow down the number of bacterial candidates to 15 (Supplementary Table S3), and to preselect strains which have a high likelihood to enhance the growth of *Actinocyclus* sp. for further experiments.

Each subsequent co-culture was established in a 70 mL tissue culture flask (Falcon) with a final volume of 50 mL (*n* = 4). For all experiments, bacteria were freshly plated from glycerol stock onto 100% Marine Agar plates 3–4 days prior to the start of the experiment and were grown from single colonies in Marine Broth. After 6 h, 100 μL of bacterial culture was transferred into fresh 100% Marine Broth and grown overnight (12 h). Bacteria were centrifuged at 1400 rpm for 6 min using an Eppendorf centrifuge 5424R. The bacterial pellet was then washed with sterile f/2 media twice, centrifuged (1,400 g for 6 min) and reconstituted in 1 mL of media. Bacteria were then diluted 1:10 in sterile media and a 200 μL aliquot was fixed with glutaraldehyde and stained with SYBR Green for enumeration *via* flow cytometry as described above. Diatoms were inoculated from 7-day old cultures into fresh media (*n* = 4) to a density of ~5,000 cells mL^−1^ and bacterial isolates were added to the axenic diatom cultures at a standardized cell density of 1,000 cells mL^−1^ (based on flow cytometry cell counts) to achieve a final ratio of 1:5 bacteria:diatom. Diatom and bacteria co-cultures were then incubated at 21°C, at 60–70 μE m^−2^ s^−1^ light intensity and gently shaken daily for 16 days. Axenic and non-axenic (culture with the whole microbiome) diatom cultures as well as bacteria in f/2 media incubated under the same conditions acted as controls (*n* = 4 for each). Over the course of the co-culture, 200 μL aliquots were sampled and fixed with glutaraldehyde (2% final concentration) every two days and both diatom and bacterial cells were quantified using flow cytometry as described above. The axenic culture controls were also checked for contamination throughout the course of the co-culture by (1) drop-plating on 100% Marine agar plates and (2) flow cytometry (following staining with SYBR green). The 15 co-cultures were run in three different batches, each with its own set of axenic and whole microbiome controls (*n* = 4).

### Statistical analyses

Statistical analyses were performed using R (v4.1.3), Excel and SPSS (v26, Armonk, NY: IBM Corp). In order to test if the diatom associated bacterial assemblages differed between time points, a Bray-Curtis matrix was built and a permutational multivariate analysis of variance (PERMANOVA) was performed using the *adonis* function from the ‘vegan’ R package (Bonferroni-Hochberg adjusted *p* < 0.05).

For the co-culture experiments, data were assessed for normality and homogeneity of variance using Shapiro and Levene’s tests before repeated-measure ANOVAs were performed. Differences in cell numbers between the co-culture and the axenic control at each time point were assessed using a Simple Main Effect test (Quinn and Keough, 2002).

To evaluate the growth-promoting effect of each bacterial strain on the diatom, specific growth rates (μ) were calculated from cell counts (cells mL^−1^) during the exponential phase (corresponding to the steepest part of the natural log-transformed cell abundance curves) of each co-culture and standard deviation of μ was calculated for the biological replicates (*n* = 4 unless otherwise indicated):


$$\mu =\frac{\ln\left(x1\right)-\ln\left(x2\right)\;}{t2-t1}$$

where *x*1 and *x*2 are the cell concentrations at time 1 (t1) and time 2 (t2).

A growth enhancement percentage from μ was also determined as: (μ_co-culture_ / μ_axenic_)/μ_co-culture_. Shapiro and Levene’s tests were used to assess the specific growth rate normality and homogeneity of variance and a one-way ANOVA followed by a Dunnett’s test (*glht* function from the ‘multicomp’ R package) were used to compare μ of every co-culture to their respective axenic controls. Additionally, we also quantified the total diatom cell concentration and the growth-promoting effect of each bacterial strain over time. To do this, we subdivided the co-cultures into three time-periods: days 0–6, days 8–12 and days 14–16 and a cell density index (CD) was then calculated for each bacterium for each of the three time-periods. Within each period, the CD index was determined by calculating the area under the curve (AUC; using the *AUC()* function from the DescTools R package) of each co-culture and dividing by the AUC of the axenic culture:


$$CD=\frac{AUC\left(co-culture\right)\;}{mean\left(AUC\left(axenic\right)\right)}$$

## Results

### *Actinocyclus* identification

The 1900 bp 18S rRNA gene sequence from the targeted diatom (Figure 1B) showed a 99.8% similarity to *Actinocyclus* sp. strain CNS00114 (Accession No. MW750345) and a 99.2% similarity to *Actinocyclus curvatulus* strain AWI 85 (Accession No. X85401). Whilst neither of these referenced strains provided suitable morphological evidence to confirm their species identification, the genus identity of the targeted strain was also confirmed by TEM to be *Actinocyclus*. Cells were cylindrical in valve view (~20–25 μm diameter), having both pseudonodulus and central annulus present, a marginal ring of labiate processes and striae that were aligned to the central stria between the fascicle edges (Figure 1A).

### The *Actinocyclus* associated bacterial community enhances diatom cell density

The presence of the *Actinocyclus* sp. microbiome increased the maximum cell concentration of the diatom by 2-fold by day 12 of the experiment, relative to the axenic control (Simple Main Effect test, *p* < 0.05, Figure 1C; Supplementary Table S4). However, the cell abundance significantly increased above the control on day 8, and remained significantly higher until the end of the experiment (day 16, Figure 1C).

**Figure 2:**
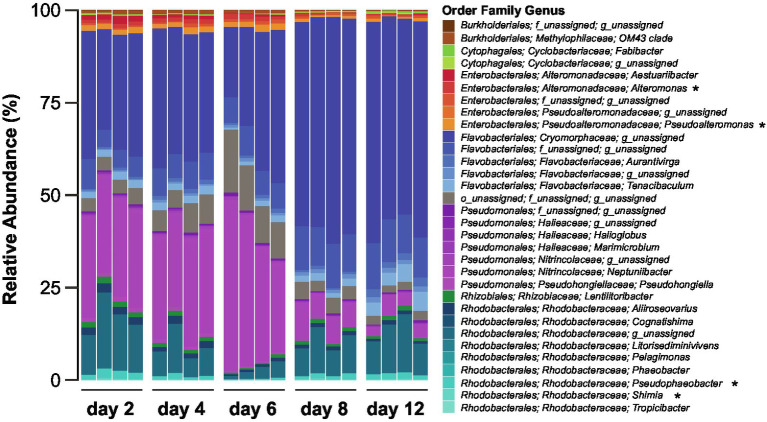
Composition of the *Actinocyclus* sp. associated bacterial communities during different diatom growth stages. Bacteria are coloured based on taxonomy and asterisks correspond to genera amongst which we have isolated bacteria used in the experiments.

The composition of the bacterial community associated with the *Actinocyclus* sp. strain, as determined by 16S rRNA gene amplicon sequencing, significantly changed over the course of the experiment (PERMANOVA and all pair-wise comparison, *p* < 0.05, Figure 2; Supplementary Table S5). A total of 638 ASVs belonging to two phyla, Bacteroidetes and Proteobacteria, were identified across all sampling times. Proteobacteria was the most abundant phylum (~55%) during the early time-points (day 2, 4 and 6), whilst the later time points (day 8 and 12) were dominated by Bacteroidetes (~75%) (Supplementary Table S6). Amongst the Proteobacteria, the relative abundance of Alphaproteobacteria (primarily Rhodobacterales) was relatively stable (~15%) throughout the diatom growth cycle, with the exception of day 6, when their relative abundance decreased (~4%). Conversely, the relative abundance of Gammaproteobacteria (i.e., Pseudomonadales and Enterobacterales) was higher during the early time points (~43%) and decreased in later time points (~11%) (Supplementary Figure S2; Supplementary Table S7). On the other hand, the Bacteroidetes (i.e., Flavobacteriales) relative abundance increased from ~42% within the first three time points to ~74% in the later ones (Supplementary Figure S2; Supplementary Table S7).

The 15 bacterial isolates chosen for co-culture experiments belonged to Alpha- and Gammaproteobacteria, including the families Rhodobacteraceae (three *Shimia*, one *Pseudophaeobacter* and three *Thalassobius*), Pseudoalteromonadaceae (two *Pseudoalteromonas*), Alteromonadaceae (one *Alteromonas*), Oceanospirillaceae (one *Marinomonas*) and Vibrionaceae (four *Vibrio*; Supplementary Table S3). Within the *Actinocyclus* sp. microbiome, six ASVs, that exactly matched the 16S rRNA gene sequences of our selected bacterial isolates, represented up to 13.7% of the communities at day 2 with an average of 8.7% across all time points (Supplementary Table S8).

### Bacterial isolates impact *Actinocyclus* specific growth rate and exponential phase

**Figure 3:**
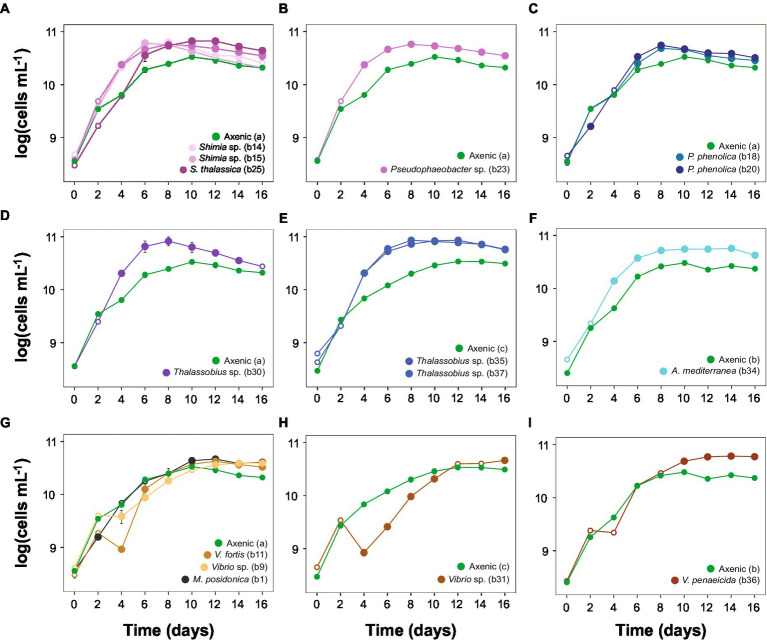
*Actinocyclus* sp. co-culture growth curves (±SEM) displaying algae log-transformed cells mL^−1^ with axenic controls. **(A-D)** and **(G)**: co-cultures with the axenic control (a); **(E**,**H)**: co-cultures with the axenic control (c); **(F**,**I)**: co-cultures with the axenic control (b). Filled circle refer to time points that were significantly different from the axenic controls (Simple Main Effect test, *p* < 0.05, Supplementary Table S4), whilst empty circles were not statistically different. Error bars represent the standard error of the mean (*n* = 4) and numbers in parenthesis in the legend correspond to bacteria strain codes. Growth curve displaying the algae log-transformed cells mL^−1^ with the whole microbiome are represented in Supplementary Figure S3 and raw data are available in Supplementary Table S10 and displayed in Supplementary Figures S4, S5.

The exponential phase of the axenic *Actinocyclus* cultures lasted for 4 days, between day 2 and 6. This length and timing were identical when *Actinocyclus* was in co-culture with 11 of the 15 bacterial strains tested (including all members of the Rhodobacteraceae, Pseudoalteromonadaceae, Alteromonadaceae and Oceanospirillaceae). However, the presence of all *Vibrio* sp. isolates delayed the onset of the exponential phase by 2 days (starting at day 4), and *Vibrio* sp. strain b31 extended the length of the phase by a further 2 days (total of 6 days) (Figure 3; Table 1).

**Table 1:** Specific growth rate and maximum cell abundance.

sample ID	Growth Rate (± SD)	GE (%)	*p* value (1)	*p* value (2)	days	batch	Max Cell Abundance (cells mL^−1^ ± SD)
Axenic	0.18 ± 0.03	/	/	< 0.001	d2 - d6	a	32,592 ± 1,439
Whole microbiome	0.36 ± 0.02	/	< 0.001	/	d2 - d8	a	79,025 ± 8,336
*Vibrio* sp. strain b9	0.17 ± 0.06	-6	1.00	< 0.001	d4 - d8	a	39,142 ± 3,080
*Pseudoalteromonas phenolica* strain b18	0.21 ± 0.01	14	0.043	< 0.001	d2 - d6	a	47,792 ± 1777
*Pseudophaeobacter* sp. strain b23	0.24 ± 0.01	25	0.002	0.001	d2 - d6	a	49,844 ± 9,344
*Marinomonas posidonica* strain b1	0.26 ± 0.01	31	0.143	0.006	d2 - d6	a	42,800 ± 1,524
*Shimia* sp. strain b14	0.28 ± 0.01	36	< 0.001	0.036	d2 - d6	a	32,983 ± 2,121
*Shimia* sp. strain b15	0.30 ± 0.01	40	< 0.001	0.199	d2 - d6	a	37,333 ± 2,291
*Pseudoalteromonas phenolica* strain b20	0.33 ± 0.01	45	< 0.001	0.839	d2 - d6	a	38,442 ± 8,785
*Shimia thalassica* strain b25	0.33 ± 0.02	45	< 0.001	0.860	d2 - d6	a	34,883 ± 1990
*Thalassobius* sp. strain b30 (*n* = 3)	0.35 ± 0.02	49	< 0.001	1.000	d2 - d6	a	33,458 ± 3,144
*Vibrio fortis* strain b11	0.36 ± 0.07	50	0.005	1.000	d4 - d8	a	48,175 ± 2,548
Axenic	0.24 ± 0.02	/	/	< 0.001	d2 - d6	b	32,850 ± 2,901
Whole microbiome	0.35 ± 0.02	/	< 0.001	/	d2 - d8	b	78,083 ± 434
*Vibrio penaeicida* strain b36	0.22 ± 0.02	-9	0.058	0.001	d4 - d8	b	29,850 ± 2,970
*Alteromonas mediterranea* strain b34	0.31 ± 0.01	23	< 0.001	0.026	d2 - d6	b	43,867 ± 1,144
Axenic	0.16 ± 0.01	/	/	< 0.001	d2 - d6	c	38,992 ± 6,014
Whole microbiome	0.41 ± 0.02	/	< 0.001	/	d2 - d8	c	121,966 ± 6,759
*Vibrio* sp. strain b31	0.23 ± 0.02	30	0.002	< 0.001	d4 - d10	c	40,150 ± 712
*Thalassobius* sp. strain b35	0.35 ± 0.01	54	< 0.001	0.004	d2 - d6	c	45,392 ± 2078
*Thalassobius* sp. strain b37	0.36 ± 0.01	56	< 0.001	0.024	d2 - d6	c	47,750 ± 3,271

Dunnett’s tests were carried out to compare the growth rates during the exponential phase between each co-culture and the corresponding axenic and whole microbiome controls (Supplementary Table S9). Days correspond to the exponential phase of each co-culture, and batch corresponds to the experiment in which each co-culture was carried. SD, standard deviation; *value of p* (1) correspond to the statistics comparing the co-cultures to the axenic controls whilst *value of p* (2) correspond to the statistics comparing the co-cultures to the whole microbiome controls; GE, Growth enhancement percentage compared to the axenic cultures.

**Figure 4:**
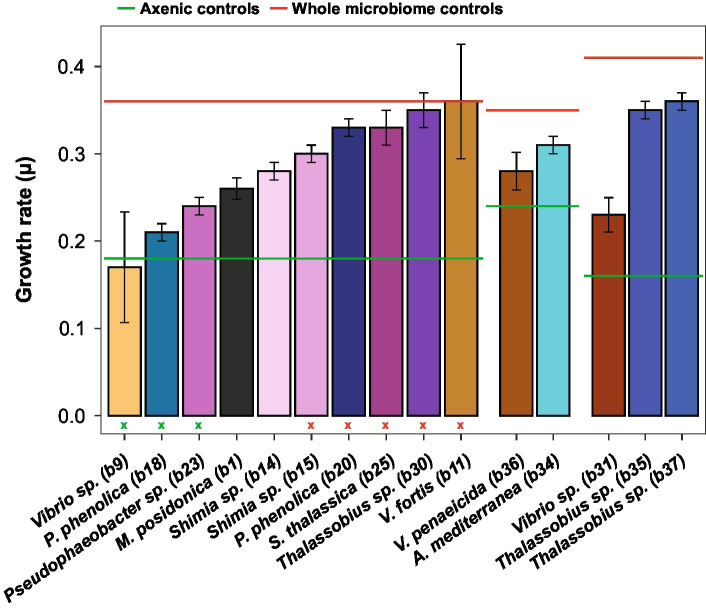
*Actinocyclus* sp. specific growth rate (± SEM) in co-culture with 15 different isolates. The six horizontal lines corresponds to the specific growth rate of the three *Actinocyclus* axenic (in green) and whole microbiome (in red) controls. Colours correspond to the different bacterial isolates. Standard error of the mean (SEM) values were calculated from 4 biological replicates except for *Thalassobius* sp. b30 (*n* = 3). Green and red (x) refer to the co-culture specific growth rate that were not significant from the axenic and the whole microbiome controls, respectively, (Dunnett’s test, *p* > 0.05, Table 1; Supplementary Table S9).

The addition of the bacterial isolates significantly affected the specific growth rate of *Actinocyclus* sp. (One-way ANOVA, F_15,55_ = 14.72, *p* < 0.001; Figure 4; Table 1; Supplementary Table S9) with 80% of the bacterial isolates enhancing the growth rate of *Actinocyclus* relative to the axenic control in the exponential phase. Overall isolates belonging to the Rhodobacteraceae enhanced the specific growth rate of *Actinocyclus* the most, with growth rates increasing by 25–56% in the presence of these bacteria, with the presence of *Thalassobius* sp. strains having the greatest effect (~ 53%). Bacterial isolates within the Vibrionaceae had less effect on *Actinocyclus* sp. specific growth rate (~16% increase) with *Vibrio* sp. strain b9 having no significant effect (Figure 4; Table 1). Interestingly, whilst the addition of most isolates resulted in a smaller growth rate compared to the cultures with the whole microbiome, the presence of three of the Rhodobacteraceae isolates (*Shimia thalassica* b25, *Shimia* sp. b15 and *Thalassobius* sp. b30), *Pseudoalteromonas phenolica* strain b20 and *Vibrio* sp. strain b11 resulted in a similar specific growth rate of *Actinocyclus* sp. to that observed in the whole microbiome cultures (Figure 4; Table 1).

Although some bacteria induced an *Actinocyclus* sp. growth rate that was similar to the cultures with their entire microbiome, this proxy alone does not adequately describe the *Actinocyclus* growth dynamics in the cultures. Indeed, *Actinocyclus* numbers in the cultures with the whole microbiome reached levels that were on average 2 times higher than the *Thalassobius* sp. b30 and *V. fortis* b11 co-cultures, and these growth enhancements were maintained for longer (Supplementary Figure S4). This observation also applies to the co-cultures, for example *Pseudophaeobacter* sp. strain b23 did not alter the diatom’s growth rate, but significantly increased cell abundance compared to the axenic control (Figures 3, 4; Table 1; Supplementary Table S4). Thus, the specific growth rate of *Actinocyclus* sp. in the presence of the different bacterial isolates was not always reflective of changes in the diatom cell abundance and does not necessarily capture the differences in growth dynamics.

### Bacteria enhance the abundance of *Actinocyclus* sp., but their effect varies based on their taxonomy

**Figure 5:**
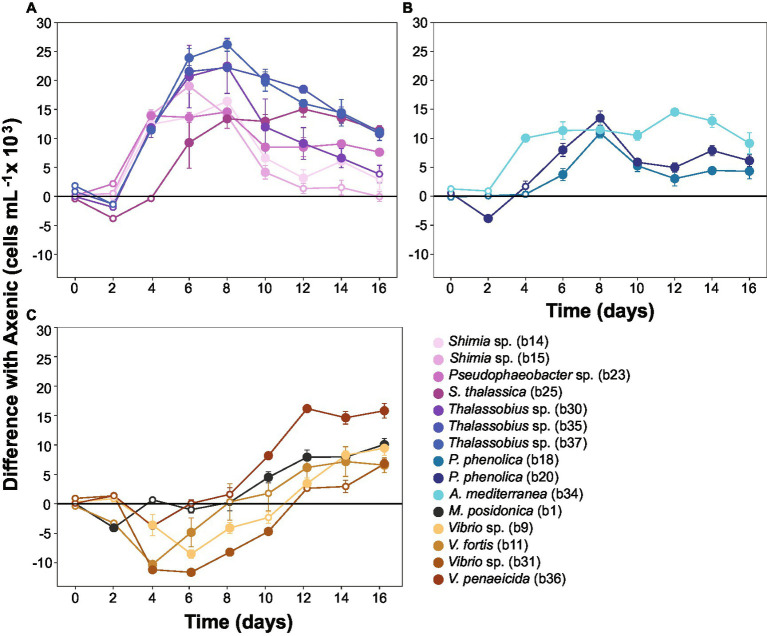
Co-cultures between *Actinocyclus* and bacteria grouped by growth dynamics. Difference between the number of cells in *Actinocyclus* sp. in co-culture with bacteria (coloured lines) and the axenic controls (black horizontal line at 0). Results for bacterial isolates belonging to **(A)**: Rhodobacteraceae; **(B)**: Pseudoalteromonadaceae and Alteromonadaceae; **(C)**: Vibrionaceae and Oceanospirillaceae. Because co-cultures were carried out in multiple batches, several axenic controls were recorded. To simplify the graphical representation here, we calculated the difference in cell counts between each co-culture and its respective axenic control (the untransformed growth curves are displayed as Figure 3 and Supplementary Figure S5). Filled circle refer to time points that were significantly different from the axenic controls (Simple Main Effect test, *p* < 0.05, Supplementary Table S4), whilst empty circles were not statistically different. Error bars represent the standard error of the mean (*n* = 4) and numbers in parenthesis in the legend correspond to bacteria strain codes. Black lines correspond to the axenic controls, note: all statistical tests were performed on the untransformed data.

To capture the multifaceted growth dynamics induced by specific bacteria in co-culture with *Actinocyclus*, we quantified differences in diatom cell abundance through time between each co-culture and the axenic controls. Each of the 15 bacterial isolates tested enhanced the abundance of *Actinocyclus* sp. during at least one time point in the co-culture experiments (Simple Main Effect test, *p* < 0.05, Supplementary Table S4). However, the magnitude and duration of these effects were highly variable between the different bacteria tested (Supplementary Figures S4, S5). Members of the Rhodobacteraceae family elicited the largest increase in diatom abundance, with 58% more cells than the control on day 8. Within the Rhodobacteraceae, *Thalassobius* sp. b37 triggered the largest increase in cell abundance, with its presence resulting in a 2-fold increase in *Actinocyclus* sp. cell numbers (Figure 5A). The increase in cell numbers caused by the Rhodobacteraceae isolates were generally sustained, with significant effects observed on average for 10 days (Simple Main Effect test, *p* < 0.05, Supplementary Table S4). In comparison, the presence of Pseudoalteromonadaceae and Alteromonadaceae had a more modest effect on *Actinocyclus* sp. growth, with only a 21% increase in diatom cell numbers observed on average (Figure 5B).

In contrast to the sustained positive effects observed amongst Rhodobacteraceae, Pseudoalteromonadaceae, and Alteromonadaceae, isolates from the Vibrionaceae and Oceanospirillaceae families only had a positive effect on the number of Actinocyclus cells during later time points of the experiment (Figure 5C). More specifically, their most pronounced effects were observed on days 14 and 16 of the experiment (28% increase in cell numbers on average), but this enhancement only lasted 3 days on average. Notably, 5 isolates belonging to the *Vibrio* and *Marinomonas* genera initially impaired diatom growth (Figure 5C). It is also worth noting that whilst some of our isolates had identical (100% match) 16S rRNA gene sequences (*Thalassobius* sp. isolates b30-b35-b37 and *Pseudoalteromonas phenolica* isolates b18-b20), they did not all induce the same growth dynamics in co-culture with *Actinocyclus* (Figure 5).

The growth enhancement observed in *Actinocyclus* sp. was part of a reciprocal interaction, because all bacterial strains tested reached significantly greater cell numbers when co-cultured with *Actinocyclus* relative to controls (Supplementary Figure S6). The only exception was *A. mediterranea*, which decreased through time when in co-culture (Supplementary Figure S6).

### Temporal variability of *Actinocyclus* cell numbers in co-cultures

**Figure 6:**
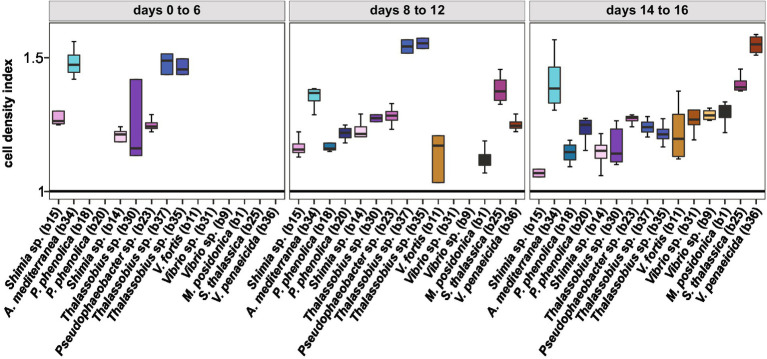
Cell density index of all bacterial isolates. Only cell density that were significant for each phase are displayed (Simple Main Effect ANOVA, *value of p* <0.05, Supplementary Table S12). Colours correspond to the different bacterial isolates. All data are available in Supplementary Table S14.

Given temporal differences in the growth enhancement effect of each bacterial isolate tested, we quantified the difference in *Actinocyclus* cell numbers between each co-culture and the axenic controls during three distinct time-period windows (day 0–6, day 8–12, and day 14–16) and calculated the growth rate and maximal cell abundance for each period (Supplementary Table S11). Our results confirmed that although all tested bacteria significantly increased the cell abundance of *Actinocyclus* in at least one of the three time periods (Simple Main Effect test, *p* < 0.05. Supplementary Table S12), their maximal effects did not occur in the same period (Supplementary Figure S7; Supplementary Table S13). Most Rhodobacteraceae and Pseudoalteromonadaceae isolates increased the diatom abundance the most between days 8–12, with a maximum cell density index of 1.6 in co-culture with *Thalassobius* sp. (b35 and b37), whilst members of the Vibrionaceae mostly increased algal cell numbers between days 14–16 (Figure 6; Supplementary Tables S13, S14).

Out of the 15 isolates tested, 6 had a 100% identity match to ASVs within the microbiome of *Actinocyclus* sp. including *Shimia* (isolates b14 and b15), *Pseudophaeobacter* (isolate b23), *Pseudoalteromonas* (isolates b18 and b20) and *Alteromonas* (isolate b34). However, although those specific ASVs were found in the *Actinocyclus* microbiome, there was no link between the timing of the growth benefit on *Actinocyclus* and the relative abundance of these bacterial isolates within the microbiome (Supplementary Figure S8).

## Discussion

A wide diversity of bacterial taxa are commonly associated with diatoms both in culture conditions (Amin et al., 2012; Ajani et al., 2018; Crenn et al., 2018; Mönnich et al., 2020) and in natural aquatic environments (Teeling et al., 2012; Arandia-Gorostidi et al., 2022). However, whilst the ecological and metabolic interactions involved in a handful of diatom-bacteria model systems have been characterised (Amin et al., 2015; Durham et al., 2015, 2017; Ferrer-González et al., 2021; Olofsson et al., 2022), the impacts of diatom-associated bacteria on host growth are still largely unknown. To gain deeper insights into the true extent of mutualistic interactions taking place within diatom microbiomes, the goal of this study was to deliver a broad-scale screening of a diatom’s associated bacteria to examine the extent and nature of beneficial partnerships across diverse bacterial associates. This analysis demonstrated that many members of the *Actinocyclus* sp. microbiome can indeed enhance the growth of the diatom host, but that their influence can vary substantially through time.

### The structure of the bacterial community associated with *Actinocyclus* sp. changes between growth stages

The *Actinocyclus* sp. bacterial community was dominated by members of the Bacteroidetes (mainly Cryomorphaceae) as well as α- and γ-Proteobacteria, which is largely consistent with the microbiome composition of other diatom species (Grossart et al., 2005; Buchan et al., 2014; Ajani et al., 2018; Stock et al., 2019; Koester et al., 2022). Our results also indicate that the *Actinocyclus* sp. bacterial community undergoes significant compositional changes throughout the diatom’s growth cycle, which is also consistent with observations in other diatom species (Moejes et al., 2017; Crenn et al., 2018). The early stages of the diatom’s growth cycle were characterised by a higher relative proportion of Proteobacteria (~55%), whilst Bacteroidetes relative abundance increased (40 to 75%) within the later time-points. Similar patterns have also been observed in various diatom species, both in culture conditions (Moejes et al., 2017), and during blooms in natural environments (Teeling et al., 2012; Needham and Fuhrman, 2016). The identity of bacteria that dominate diatom microbiomes at a given time may be strongly influenced by the photosynthates exuded by the diatom, which are known to differ between growth stages (Barofsky et al., 2009; Sarmento et al., 2013; Olofsson et al., 2022). For example, cells in exponential phase and later stages tend to actively release a higher proportion of high molecular weight compounds (e.g., carbohydrates such as polysaccharides) (Myklestad, 1974; Biddanda and Benner, 1997; Granum et al., 2002), which Flavobacteria are known to effectively metabolise (Cottrell and Kirchman, 2000; Buchan et al., 2014). Members of Flavobacteriaceae effectively remineralise organic matter (Kirchman, 2002), which may explain why they accounted for a larger proportion of the bacterial community at later growth stages, when nutrients (N, Si, P) become limited. It is therefore likely that the shift from a dominance of Proteobacteria during the early growth phases of *Actinocyclus*, to Flavobacteria during later stages, is governed by a changing pool of chemicals released by the diatom.

Interestingly, peaks in the relative abundance of ASVs within the *Actinocyclus* microbiome that were identical to the tested isolates did not always align with the timing of the growth benefits observed in co-cultures (Supplementary Figure S8). Although these isolates demonstrated beneficial effects on *Actinocyclus’s* growth in co-culture, it is possible that interactions between multiple members of the microbiome may further influence the growth benefit observed in the whole microbiome cultures.

### Different bacteria have different growth promoting effect through time

Consistent with observations in other diatom species (Grossart et al., 2005; Sison-Mangus et al., 2014; Windler et al., 2014; Amin et al., 2015), we found that when in presence of its full bacterial consortium, the abundance of *Actinocyclus* sp. was double that of the axenic cultures, emphasising the strong positive influence that bacteria can have on diatom growth (Amin et al., 2012). This was reinforced by the observation that most of the bacterial isolates enhanced the specific growth rate of the diatom with several growing as fast as the cultures with the whole microbiome. However, although the overall growth response of *Actinocyclus* to the different bacterial isolates tested here was almost entirely positive, none of them reached the maximum cell abundance of the cultures containing the full microbiome, which emphasises the cumulative effect of the microbiome as a whole. Interestingly, even if the maximum cell numbers reached or the specific growth rate between the controls and the co-cultures were similar, the difference in cell abundance through the growth of the diatom significantly changed and was bacteria-specific. This is consistent with a recent study which demonstrated that within 256 diatom-bacteria dual co-cultures (in well-plates), multiple bacterial isolates had different effects on the growth of the 8 diatoms tested and this effect was variable depending on the growth stage of the algae (Deng et al., 2022).

In general, strains belonging to the Rhodobacteraceae had the strongest positive impact on *Actinocyclus* sp. cell numbers (especially *Thalassobius sp*.), with greatest levels of growth promotion occurring between days 8 to 12. This concurs with various studies that have shown that members of the Rhodobacteraceae can enhance algal growth (Amin et al., 2015; Durham et al., 2015; Johansson et al., 2019). For example, *Sulfitobacter* sp. increases cell division in the diatom *Pseudo-nitzschia multiseries*, through the production of the plant growth-promoting hormone indole-3-acetic acid (IAA) (Amin et al., 2015), and *Ruegeria pomeroyi* enhances the growth of *Thalassiosira pseudonana,* through the provision of vitamin B_12_ (Durham et al., 2015). On the other hand, most of the strains belonging to Gammaproteobacteria, including members of the *Alteromonas*, *Pseudoalteromonas*, *Vibrio* and *Marinomonas* genera, had the greatest effect on *Actinocyclus* sp. cell numbers during days 14 to 16. Whilst members of the Alteromonadaceae and Vibrionaceae family are often attracted to diatom metabolites (Sarmento et al., 2013; Taylor and Cunliffe, 2017; Raina et al., 2022), they have been demonstrated elsewhere to have negative or neutral effects on diatom growth (Kim et al., 1999; Aharonovich and Sher, 2016; Wang et al., 2016). However, *Alteromonas* bacteria can have a positive effect on diatom growth and possess genes involved in vitamin synthesis (B_1_, B_7_ and B_12_) and degradation of hydrogen peroxide (catalase enzyme), which can reduce oxidative stress related to photosynthesis (Garcia et al., 2017; Baker and Kemp, 2020). Additionally, recent whole-genome analyses also reported that many Gammaproteobacteria (i.e., *Alteromonas*, *Pseudoalteromonas* and *Marinomonas* genera) possess genes involved in phytoplankton-bacterial interactions, such as the production of vitamin B_1_, plant growth promoting hormones or siderophores, suggesting that they may also promote diatom growth (Zoccarato et al., 2022).

The time-dependent effects reported here could indeed be explained by the provision of different molecules by bacterial associates. In culture during early growth phases, diatom cells are not likely to be limited in vitamins or trace metals, but they could benefit from the production of specific secondary metabolites such as growth-promoting hormones. Towards later growth-phases, bacterial provision of remineralized nutrients (e.g., phosphate, nitrogen), as well as trace metals and vitamins that would become limited in the culture medium (Christie-Oleza et al., 2015, 2017), may contribute to enhanced diatom growth. In addition, diatoms may also benefit from bacteria through the removal of toxic compounds that build-up in later growth stages, such as hydrogen peroxide (H_2_O_2_) (Morris et al., 2011; Coe et al., 2016). Whilst further analyses, coupling chemical and biological measurements will be required to tease-apart the suite of specific mechanisms likely responsible for the time-dependent impact of different bacteria on diatom growth, our results provide an intriguing new insight into the multifaceted influence of diatom associated bacterial communities on the growth of their hosts.

## Conclusion

Whilst there is mounting evidence that diatoms develop important ecological associations with specific bacterial partners, the microbiome of diatoms is generally composed of a wide diversity of bacteria, making it difficult to identify the role played by individual bacterial species. Here, we examined the effect of 15 different bacteria associated with *Actinocyclus* sp. on the growth of this diatom. Whilst all 15 tested bacteria enhanced *Actinocyclus* sp. growth compared to the controls, their effects differed in magnitude, duration and timing. Our results not only demonstrate that diatom growth can be enhanced by multiple members of the host’s associated bacterial community, but that the beneficial impacts of different bacteria vary according to time. These observations provide further evidence for the important role of bacteria in defining the ecology and fitness of diatoms and the complex nature of these relationships.

## Data availability statement

The datasets presented in this study can be found in online repositories. The names of the repository/repositories and accession number(s) can be found in the article/Supplementary material. Raw Illumina reads of bacterial isolates and microbiome data (16S rRNA) are available in NCBI Sequence Read Archive (SRA) under the BioProject PRJNA885573. Sanger sequences of the bacterial isolates (16S rRNA) and Actinocyclus sp. (18S rRNA) are available in GenBank under the accession numbers OP558536-OP558550 and OP566387. The analysis script for Illumina sequences processing is available from GitHub (https://github.com/NineFR09/Frontiers-10.3389-fmicb.2023.12-Growth-enhancing-effect-of-Actinocyclus-diatom-associated-bacteria.git).

## Author contributions

NR, AB, JS, J-BR, and, PA designed all the experiments and wrote the manuscript and all authors edited the manuscript and agree to the published version of the manuscript. AB isolated the diatom. NR performed all the experiments except the reseeding experiment performed by AK. NR processed all data. NR and AB generated and analysed the microbiome and bacterial isolate amplicon data. All authors contributed to the article and approved the submitted version.

## Funding

This research was supported by an Australian Research Council (ARC) Discovery Project DP180100838 awarded to JS, J-BR and PA.

## Conflict of interest

The authors declare that the research was conducted in the absence of any commercial or financial relationships that could be construed as a potential conflict of interest.

## Publisher’s note

All claims expressed in this article are solely those of the authors and do not necessarily represent those of their affiliated organizations, or those of the publisher, the editors and the reviewers. Any product that may be evaluated in this article, or claim that may be made by its manufacturer, is not guaranteed or endorsed by the publisher.

## Footnotes

^1^https://imagej.net/ImageJ1

## References

[ref1] Aharonovich D. Sher D. (2016). Transcriptional response of Prochlorococcus to co-culture with a marine Alteromonas: differences between strains and the involvement of putative infochemicals. ISME J. 10, 2892–2906. doi: 10.1038/ismej.2016.70, PMID: 27128996 PMC5148192

[ref2] Ajani P. A. Kahlke T. Siboni N. Carney R. Murray S. A. Seymour J. R. (2018). The microbiome of the cosmopolitan diatom leptocylindrus reveals significant spatial and temporal variability. Front. Microbiol. 9, 1–12. doi: 10.3389/fmicb.2018.02758, PMID: 30498485 PMC6249420

[ref3] Amin S. A. Green D. H. Hart M. C. Kupper F. C. Sunda W. G. Carrano C. J. (2009). Photolysis of iron-siderophore chelates promotes bacterial-algal mutualism. Proc. Natl. Acad. Sci. 106, 17071–17076. doi: 10.1073/pnas.0905512106, PMID: 19805106 PMC2761308

[ref4] Amin S. A. Hmelo L. R. Van Tol H. M. Durham B. P. Carlson L. T. Heal K. R.. (2015). Interaction and signalling between a cosmopolitan phytoplankton and associated bacteria. Nature 522, 98–101. doi: 10.1038/nature14488, PMID: 26017307

[ref5] Amin S. A. Parker M. S. Armbrust E. V. (2012). Interactions between diatoms and Bacteria. Microbiol. Mol. Biol. Rev. 76, 667–684. doi: 10.1128/mmbr.00007-12, PMID: 22933565 PMC3429620

[ref6] Andersen R. A. Berges J. A. Harrison P. J. Watanabe M. M. (2005). Recipes for freshwater and seawater media. Algal Cult. Tech., 429–538. doi: 10.1016/b978-012088426-1/50027-5

[ref7] Arandia-Gorostidi N. Krabberød A. K. Logares R. Deutschmann I. M. Scharek R. Morán X. A. G.. (2022). Novel interactions between phytoplankton and Bacteria shape microbial seasonal dynamics in Coastal Ocean waters. Front. Mar. Sci. 9:901201. doi: 10.3389/fmars.2022.901201

[ref8] Azam F. Malfatti F. (2007). Microbial structuring of marine ecosystems. Nat. Rev. Microbiol. 5, 782–791. doi: 10.1038/nrmicro1747 17853906

[ref9] Baker L. J. Kemp P. F. (2020). Bacterial inoculations can perturb the growth trajectory of diatoms with an existing microbiome. PeerJ 8, e8352–e8320. doi: 10.7717/peerj.8352, PMID: 32025366 PMC6991125

[ref10] Barak-gavish N. Dassa B. Kuhlisch C. Nussbaum I. Rosenberg G. (2022). Bacterial lifestyle switch in response to algal metabolites. Elife 24, 1–34. doi: 10.7554/eLife.84400. PMC9873259 36691727

[ref11] Barofsky A. Vidoudez C. Pohnert G. (2009). Metabolic profiling reveals growth stage variability in diatom exudates. Limnol. Oceanogr. Methods 7, 382–390. doi: 10.4319/lom.2009.7.382

[ref12] Behringer G. Ochsenkühn M. A. Fei C. Fanning J. Koester J. A. Amin S. A. (2018). Bacterial communities of diatoms display strong conservation across strains and time. Front. Microbiol. 9, 1–15. doi: 10.3389/fmicb.2018.00659, PMID: 29681892 PMC5897529

[ref13] Biddanda B. Benner R. (1997). Carbon, nitrogen, and carbohydrate fluxes during the production of particulate and dissolved organic matter by marine phytoplankton. Limnol. Oceanogr. 42, 506–518. doi: 10.4319/lo.1997.42.3.0506

[ref14] Bramucci A. R. Labeeuw L. Orata F. D. Ryan E. M. Malmstrom R. R. Case R. J. (2018). The bacterial symbiont *Phaeobacter inhibens* shapes the life history of its algal host *Emiliania huxleyi*. Front. Mar. Sci. 5:188. doi: 10.3389/fmars.2018.00188

[ref15] Brown M. V. Van De Kamp J. Ostrowski M. Seymour J. R. Ingleton T. Messer L. F.. (2018). Data descriptor: systematic, continental scale temporal monitoring of marine pelagic microbiota by the Australian marine microbial biodiversity initiative. Sci. Data 5, 1–10. doi: 10.1038/sdata.2018.130, PMID: 30015804 PMC6049030

[ref16] Buchan A. LeCleir G. R. Gulvik C. A. González J. M. (2014). Master recyclers: features and functions of bacteria associated with phytoplankton blooms. Nat. Rev. Microbiol. 12, 686–698. doi: 10.1038/nrmicro3326, PMID: 25134618

[ref17] Callahan B. J. Wong J. Heiner C. Oh S. Theriot C. M. Gulati A. S.. (2019). High-throughput amplicon sequencing of the full-length 16S rRNA gene with single-nucleotide resolution. Nucleic Acids Res. 47:e103. doi: 10.1093/nar/gkz569, PMID: 31269198 PMC6765137

[ref18] Christie-Oleza J. A. Scanlan D. J. Armengaud J. (2015). “You produce whilst I clean up”, a strategy revealed by exoproteomics during Synechococcus-Roseobacter interactions. Proteomics 15, 3454–3462. doi: 10.1002/pmic.201400562, PMID: 25728650 PMC4949626

[ref19] Christie-Oleza J. A. Sousoni D. Lloyd M. Armengaud J. Scanlan D. J. (2017). Nutrient recycling facilitates long-term stability of marine microbial phototroph-heterotroph interactions. Nat. Microbiol. 2:17100. doi: 10.1038/nmicrobiol.2017.100, PMID: 28650444 PMC5495174

[ref20] Cirri E. Pohnert G. (2019). Algae−bacteria interactions that balance the planktonic microbiome. New Phytol. 223, 100–106. doi: 10.1111/nph.15765, PMID: 30825329

[ref21] Coe A. Ghizzoni J. LeGault K. Biller S. Roggensack S. E. Chisholm S. W. (2016). Survival of Prochlorococcus in extended darkness. Limnol. Oceanogr. 61, 1375–1388. doi: 10.1002/lno.10302

[ref22] Cooper M. B. Kazamia E. Helliwell K. E. Kudahl U. J. Sayer A. Wheeler G. L.. (2019). Cross-exchange of B-vitamins underpins a mutualistic interaction between Ostreococcus tauri and *Dinoroseobacter shibae*. ISME J. 13, 334–345. doi: 10.1038/s41396-018-0274-y, PMID: 30228381 PMC6331578

[ref23] Cottrell M. T. Kirchman D. L. (2000). Natural assemblages of marine Proteobacteria and members of the Cytophaga-Flavobacter cluster consuming low- and high-molecular-weight dissolved organic matter. Available at: https://journals.asm.org/journal/aem. 10.1128/aem.66.4.1692-1697.2000 PMC92043 10742262

[ref24] Crenn K. Duffieux D. Jeanthon C. (2018). Bacterial Epibiotic communities of ubiquitous and abundant marine diatoms are distinct in short- and long-term associations. Front. Microbiol. 9, 1–12. doi: 10.3389/fmicb.2018.02879, PMID: 30564203 PMC6288172

[ref25] Croft M. T. Lawrence A. D. Raux-Deery E. Warren M. J. Smith A. G. (2005). Algae acquire vitamin B12 through a symbiotic relationship with bacteria. Nature 438, 90–93. doi: 10.1038/nature04056, PMID: 16267554

[ref26] Deng Y. Mauri M. Vallet M. Staudinger M. Allen R. J. Pohnert G. (2022). Dynamic diatom-Bacteria consortia in synthetic plankton communities. Appl. Environ. Microbiol. 88:e0161922. doi: 10.1128/aem.01619-22, PMID: 36300970 PMC9680611

[ref27] Durham B. P. Dearth S. P. Sharma S. Amin S. A. Smith C. B. Campagna S. R.. (2017). Recognition cascade and metabolite transfer in a marine bacteria-phytoplankton model system. Environ. Microbiol. 19, 3500–3513. doi: 10.1111/1462-2920.13834, PMID: 28631440

[ref28] Durham B. P. Van Mooy B. A. S. Moran M. A. Amin S. A. Luo H. Armbrust E. V.. (2015). Cryptic carbon and sulfur cycling between surface ocean plankton. Proc. Natl. Acad. Sci. 112, 453–457. doi: 10.1073/pnas.1413137112, PMID: 25548163 PMC4299198

[ref29] Falkowski P. G. Barber R. T. Smetacek V. (1998). Biogeochemical controls and feedbacks on ocean primary production. Science 281, 200–207. doi: 10.1126/science.281.5374.200, PMID: 9660741

[ref30] Falkowski P. G. Fenchel T. Delong E. F. (2008). The microbial engines that drive earth’s biogeochemical cycles. Science 320, 1034–1039. doi: 10.1126/science.1153213 18497287

[ref31] Ferrer-González F. X. Widner B. Holderman N. R. Glushka J. Edison A. S. Kujawinski E. B.. (2021). Resource partitioning of phytoplankton metabolites that support bacterial heterotrophy. ISME J. 15, 762–773. doi: 10.1038/s41396-020-00811-y, PMID: 33097854 PMC8027193

[ref32] Field C. B. Behrenfeld M. J. Randerson J. T. Falkowski P. (1998). Primary production of the biosphere: integrating terrestrial and oceanic components. Science 281, 237–240. doi: 10.1126/science.281.5374.237, PMID: 9657713

[ref33] Garcia N. S. Yung C. M. Davis K. M. Rynearson T. Hunt D. E. (2017). Draft genome sequences of three bacterial isolates from cultures of the marine diatom *Thalassiosira rotula*. Genome Announc. 5:17. doi: 10.1128/genomeA.00316-17, PMID: 28473397 PMC5442376

[ref34] Gärdes A. Ramaye Y. Grossart H. P. Passow U. Ullrich M. S. (2012). Effects of *Marinobacter adhaerens* HP15 on polymer exudation by *Thalassiosira weissflogii* at different N:P ratios. Mar. Ecol. Prog. Ser. 461, 1–14. doi: 10.3354/meps09894

[ref35] Granum E. Kirkvold S. Myklestad S. M. (2002). Cellular and extracellular production of carbohydrates and amino acids by the marine diatom *Skeletonema costatum*: diel variations and effects of N depletion. Mar. Ecol. Prog. Ser. 242, 83–94. doi: 10.3354/meps242083

[ref36] Grossart H. P. Levold F. Allgaier M. Simon M. Brinkhoff T. (2005). Marine diatom species harbour distinct bacterial communities. Environ. Microbiol. 7, 860–873. doi: 10.1111/j.1462-2920.2005.00759.x, PMID: 15892705

[ref37] Harvey E. L. Deering R. W. Rowley D. C. Gamal A. El Schorn M. Moore B. S.. (2016). A bacterial quorum-sensing precursor induces mortality in the marine coccolithophore, *Emiliania huxleyi*. Front. Microbiol. 7, 1–12. doi: 10.3389/fmicb.2016.00059, PMID: 26870019 PMC4737879

[ref9001] Hasle G. R. Fryxell G. A. (1970). Diatoms: Cleaning and mounting for light and electron microscopy. Trans Am Microsc Soc. 89, 469–474.

[ref9002] Hasle G. R. Syvertsen E. E. (1996). Marine diatoms. Identifying Marine Phytoplankton. ed. Tomas C. R. (San Diego: Academic Press).

[ref38] Johansson O. N. Pinder M. I. M. Ohlsson F. Egardt J. Töpel M. Clarke A. K. (2019). Friends with benefits: exploring the Phycosphere of the marine diatom Skeletonema marinoi. Front. Microbiol. 10, 1–11. doi: 10.3389/fmicb.2019.01828, PMID: 31447821 PMC6691348

[ref39] Kim J. H. Park J. H. Song Y. H. Cahng D. S. (1999). Isolation and characterization of the marine bacterium, Alteromonas sp. SR-14 inhibiting the growth of diatom, Chaetoceros species. J. Korean Fish. Soc. 32, 155–159.

[ref40] Kirchman D. L. (2002). The ecology of Cytophaga-Flavobacteria in aquatic environments. FEMS Microbiol. Ecol. 39, 91–100. doi: 10.1111/j.1574-6941.2002.tb00910.x, PMID: 19709188

[ref41] Koester I. Quinlan Z. A. Nothias L. White M. E. Rabines A. Petras D.. (2022). Illuminating the dark metabolome of pseudo-nitzschia -microbiome associations. Environ. Microbiol. 24, 5408–5424. doi: 10.1111/1462-2920.16242, PMID: 36222155 PMC9707391

[ref42] Lafond A. Leblanc K. Legras J. Cornet V. Quéguiner B. (2020). The structure of diatom communities constrains biogeochemical properties in surface waters of the Southern Ocean (Kerguelen plateau). J. Mar. Syst. 212:103458. doi: 10.1016/j.jmarsys.2020.103458

[ref43] Larsson M. E. Bramucci A. R. Collins S. Hallegraeff G. Kahlke T. Raina J. B.. (2022). Mucospheres produced by a mixotrophic protist impact ocean carbon cycling. Nat. Commun. 13, 1301–1315. doi: 10.1038/s41467-022-28867-8, PMID: 35288549 PMC8921327

[ref44] Le Reun N. Bramucci A. O’brien J. Ostrowski M. Brown M. V. Van De Kamp J.. (2022). Diatom biogeography, temporal dynamics, and links to Bacterioplankton across seven oceanographic time-series sites spanning the Australian continent. Microorganisms 10:20338. doi: 10.3390/microorganisms10020338, PMID: 35208793 PMC8880096

[ref45] Liu Y. Debeljak P. Rembauville M. Blain S. Obernosterer I. (2019). Diatoms shape the biogeography of heterotrophic prokaryotes in early spring in the Southern Ocean. Environ. Microbiol. 21, 1452–1465. doi: 10.1111/1462-2920.14579, PMID: 30834642

[ref46] Luria C. M. Amaral-Zettler L. A. Ducklow H. W. Rich J. J. (2016). Seasonal succession of free-living bacterial communities in coastal waters of the western antarctic peninsula. Front. Microbiol. 7, 1–13. doi: 10.3389/fmicb.2016.01731, PMID: 27857708 PMC5093341

[ref47] Malviya S. Scalco E. Audic S. Vincent F. Veluchamy A. Poulain J.. (2016). Insights into global diatom distribution and diversity in the world’s ocean. Proc. Natl. Acad. Sci. 113, E1516–E1525. doi: 10.1073/pnas.1509523113, PMID: 26929361 PMC4801293

[ref48] Moejes F. Succurro A. Popa O. Maguire J. Ebenhöh O. (2017). Dynamics of the bacterial community associated with *Phaeodactylum tricornutum* cultures. PRO 5:77. doi: 10.3390/pr5040077

[ref49] Mönnich J. Tebben J. Bergemann J. Case R. Wohlrab S. Harder T. (2020). Niche-based assembly of bacterial consortia on the diatom *Thalassiosira rotula* is stable and reproducible. ISME J. 14, 1614–1625. doi: 10.1038/s41396-020-0631-5, PMID: 32203123 PMC7242391

[ref50] Morris J. J. Johnson Z. I. Szul M. J. Keller M. Zinser E. R. (2011). Dependence of the cyanobacterium Prochlorococcus on hydrogen peroxide scavenging microbes for growth at the ocean’s surface. PLoS One 6:e16805. doi: 10.1371/journal.pone.0016805, PMID: 21304826 PMC3033426

[ref51] Myklestad S. (1974). Production of carbohydrates by marine planktonic diatoms. I. Comparison of nine different species in culture. J. Exp. Mar. Biol. Ecol. 15, 261–274. doi: 10.1016/0022-0981(74)90049-5

[ref52] Needham D. M. Fuhrman J. A. (2016). Pronounced daily succession of phytoplankton, archaea and bacteria following a spring bloom. Nat. Microbiol. 1:16005. doi: 10.1038/nmicrobiol.2016.5, PMID: 27572439

[ref53] Olofsson M. Ferrer-gonzález F. X. Uchimiya M. Smith C. B. Edison A. S. Moran M. A. (2022). Growth-stage-related shifts in diatom endometabolome composition set the stage for bacterial heterotrophy. ISME COMMUN 2:28. doi: 10.1038/s43705-022-00116-5 PMC9723723 37938663

[ref54] Paulson J. N. Colin Stine O. Bravo H. C. Pop M. (2013). Differential abundance analysis for microbial marker-gene surveys. Nat. Methods 10, 1200–1202. doi: 10.1038/nmeth.2658, PMID: 24076764 PMC4010126

[ref55] Quinn G. P. Keough M. J. (2002). Experimental design and data analysis for biologists. Cambridge Univ. Press. Engalnd.

[ref56] Raina J. B. Lambert B. S. Parks D. H. Rinke C. Siboni N. Bramucci A.. (2022). Chemotaxis shapes the microscale organization of the ocean’s microbiome. Nature 605, 132–138. doi: 10.1038/s41586-022-04614-3, PMID: 35444277

[ref57] Sapp M. Wichels A. Gerdts G. (2007). Impacts of cultivation of marine diatoms on the associated bacterial community. Appl. Environ. Microbiol. 73, 3117–3120. doi: 10.1128/AEM.02274-06, PMID: 17369346 PMC1892871

[ref58] Sarmento H. Romera-Castillo C. Lindh M. Pinhassi J. Sala M. M. Gasol J. M.. (2013). Phytoplankton species-specific release of dissolved free amino acids and their selective consumption by bacteria. Limnol. Oceanogr. 58, 1123–1135. doi: 10.4319/lo.2013.58.3.1123

[ref9003] Schmidt A. (1878). Atlas der Diatomaceen-kunde Series II: Heft 15: Aschersleben: Verlag von Ernst Schlegel. 57–60.

[ref59] Seyedsayamdost M. R. Carr G. Kolter R. Clardy J. (2011a). Roseobacticides: small molecule modulators of an algal-bacterial symbiosis. J. Am. Chem. Soc. 133, 18343–18349. doi: 10.1021/ja207172s, PMID: 21928816 PMC3211371

[ref60] Seyedsayamdost M. R. Case R. J. Kolter R. Clardy J. (2011b). The Jekyll-and-Hyde chemistry of *phaeobacter gallaeciensis*. Nat. Chem. 3, 331–335. doi: 10.1038/nchem.1002, PMID: 21430694 PMC3376411

[ref61] Seymour J. R. Amin S. A. Raina J. B. Stocker R. (2017). Zooming in on the phycosphere: the ecological interface for phytoplankton-bacteria relationships. Nat. Microbiol. 2:65. doi: 10.1038/nmicrobiol.2017.65 28555622

[ref62] Shibl A. A. Isaac A. Ochsenkühn M. A. Cárdenas A. Fei C. Behringer G.. (2020). Diatom modulation of select bacteria through use of two unique secondary metabolites. Proc. Natl. Acad. Sci. 117, 27445–27455. doi: 10.1073/pnas.2012088117, PMID: 33067398 PMC7959551

[ref63] Shishlyannikov S. M. Zakharova Y. R. Volokitina N. A. Mikhailov I. S. Petrova D. P. Likhoshway Y. V. (2011). A procedure for establishing an axenic culture of the diatom *Synedra acus* subsp. radians (Kütz.) Skabibitsch. From Lake Baikal. Limnol. Oceanogr. Methods 9, 478–484. doi: 10.4319/lom.2011.9.478

[ref64] Sison-Mangus M. P. Jiang S. Kudela R. M. Mehic S. (2016). Phytoplankton-associated bacterial community composition and succession during toxic diatom bloom and non-bloom events. Front. Microbiol. 7, 1–12. doi: 10.3389/fmicb.2016.01433, PMID: 27672385 PMC5018474

[ref65] Sison-Mangus M. P. Jiang S. Tran K. N. Kudela R. M. (2014). Host-specific adaptation governs the interaction of the marine diatom, pseudo-nitzschia and their microbiota. ISME J. 8, 63–76. doi: 10.1038/ismej.2013.138, PMID: 23985747 PMC3869016

[ref66] Stock W. Blommaert L. De Troch M. Mangelinckx S. Willems A. Vyverman W.. (2019). Host specificity in diatom-bacteria interactions alleviates antagonistic effects. FEMS Microbiol. Ecol. 95:fiz171. doi: 10.1093/femsec/fiz171, PMID: 31647551

[ref67] Taylor J. D. Cunliffe M. (2017). Coastal bacterioplankton community response to diatom-derived polysaccharide microgels. Environ. Microbiol. Rep. 9, 151–157. doi: 10.1111/1758-2229.12513, PMID: 27943607

[ref68] Teeling H. Fuchs B. M. Becher D. Klockow C. Gardebrecht A. Bennke C. M.. (2012). Substrate-controlled succession of marine bacterioplankton populations induced by a phytoplankton bloom. Science 336, 608–611. doi: 10.1126/science.1218344, PMID: 22556258

[ref69] Wang H. Butt L. Rooks P. Khan F. Allen M. J. Ali S. T. (2016). Characterisation of algicidal bacterial exometabolites against the lipid-accumulating diatom Skeletonema sp. Algal Res. 13, 1–6. doi: 10.1016/j.algal.2015.11.012

[ref70] Wang Q. Garrity G. M. Tiedje J. M. Cole J. R. (2007). Naïve Bayesian classifier for rapid assignment of rRNA sequences into the new bacterial taxonomy. Appl. Environ. Microbiol. 73, 5261–5267. doi: 10.1128/AEM.00062-07, PMID: 17586664 PMC1950982

[ref71] Windler M. Bova D. Kryvenda A. Straile D. Gruber A. Kroth P. G. (2014). Influence of bacteria on cell size development and morphology of cultivated diatoms. Phycol. Res. 62, 269–281. doi: 10.1111/pre.12059

[ref72] Worden A. Z. Follows M. J. Giovannoni S. J. Wilken S. Zimmerman A. E. Keeling P. J. (2015). Rethinking the marine carbon cycle: factoring in the multifarious lifestyles of microbes. Science 347:347. doi: 10.1126/science.1257594, PMID: 25678667

[ref73] Yilmaz P. Parfrey L. W. Yarza P. Gerken J. Ludwig W. Pruesse E.. (2014). The SILVA and all-species living tree project (LTP) taxonomic frameworks. Nucleic Acids Res. 42, D643–D648. doi: 10.1093/nar/gkt1209, PMID: 24293649 PMC3965112

[ref74] Zoccarato L. Sher D. Miki T. Segrè D. Grossart H. P. (2022). A comparative whole-genome approach identifies bacterial traits for marine microbial interactions. Commun. Biol. 5, 276–213. doi: 10.1038/s42003-022-03184-4, PMID: 35347228 PMC8960797

