# Supplementary material for: Temporal variability in the growth-enhancing effects of different bacteria within the microbiome of the diatom Actinocyclus sp
Source: Front Microbiol. 2023 Aug 7;14:1230349. doi: 10.3389/fmicb.2023.1230349 (PMC10440540; doi:10.3389/fmicb.2023.1230349)
Supplement: Supplementary file 2 [file Data_Sheet_2.pdf]

Supplementary Figures and Tables:

Temporal variability in the growth-enhancing effects of different bacteria within the microbiome of the diatom *Actinocyclus* sp.

Nine Le Reun[1]*, Anna Bramucci[1], Penelope Ajani[2], Abeeha Khalil[1], Jean-Baptiste Raina[1] and Justin R. Seymour[1]

[1] Climate Change Cluster, University of Technology Sydney (UTS), 2007, NSW, Australia

[2] School of Life Sciences, University of Technology Sydney, Ultimo, NSW 2007, Australia

* Correspondence: nine09@outlook.fr

Contents:
Supplementary Figures S1, S2, S3, S4, S5, S6, S7, S8

As Separate Excel Files:
Supplementary Tables S1, S2, S3, S4, S5, S6, S7, S8, S9, S10, S11, S12, S13, S14, S15, S16,S17

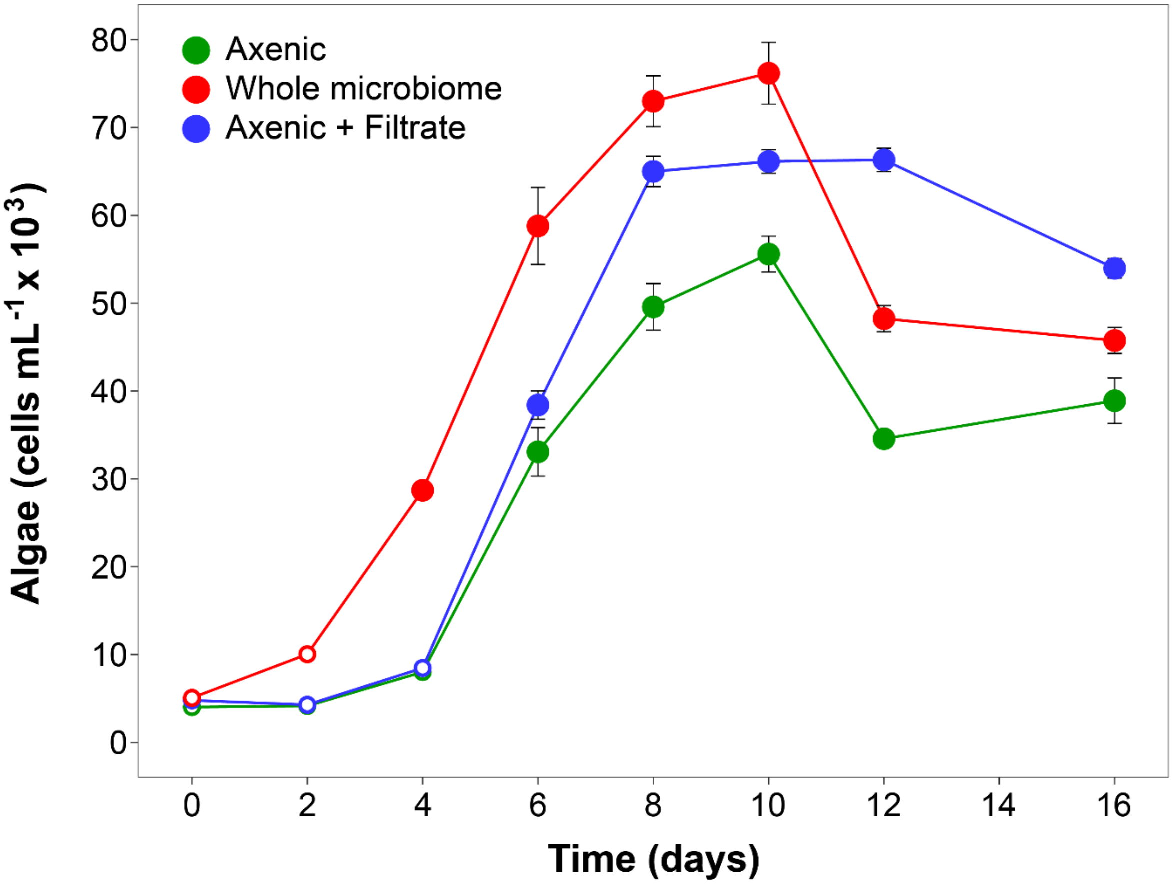

Figure S1: ***Actinocyclus*** **sp. reseeding experiment**. Filled circles refer to time points that were significantly different from the axenic controls (Simple Main Effect Test, $p < 0.05$, Table S15), while empty circles were not statistically different. Green lines correspond to the axenic control. Error bars represent the standard error of the mean (n=4).

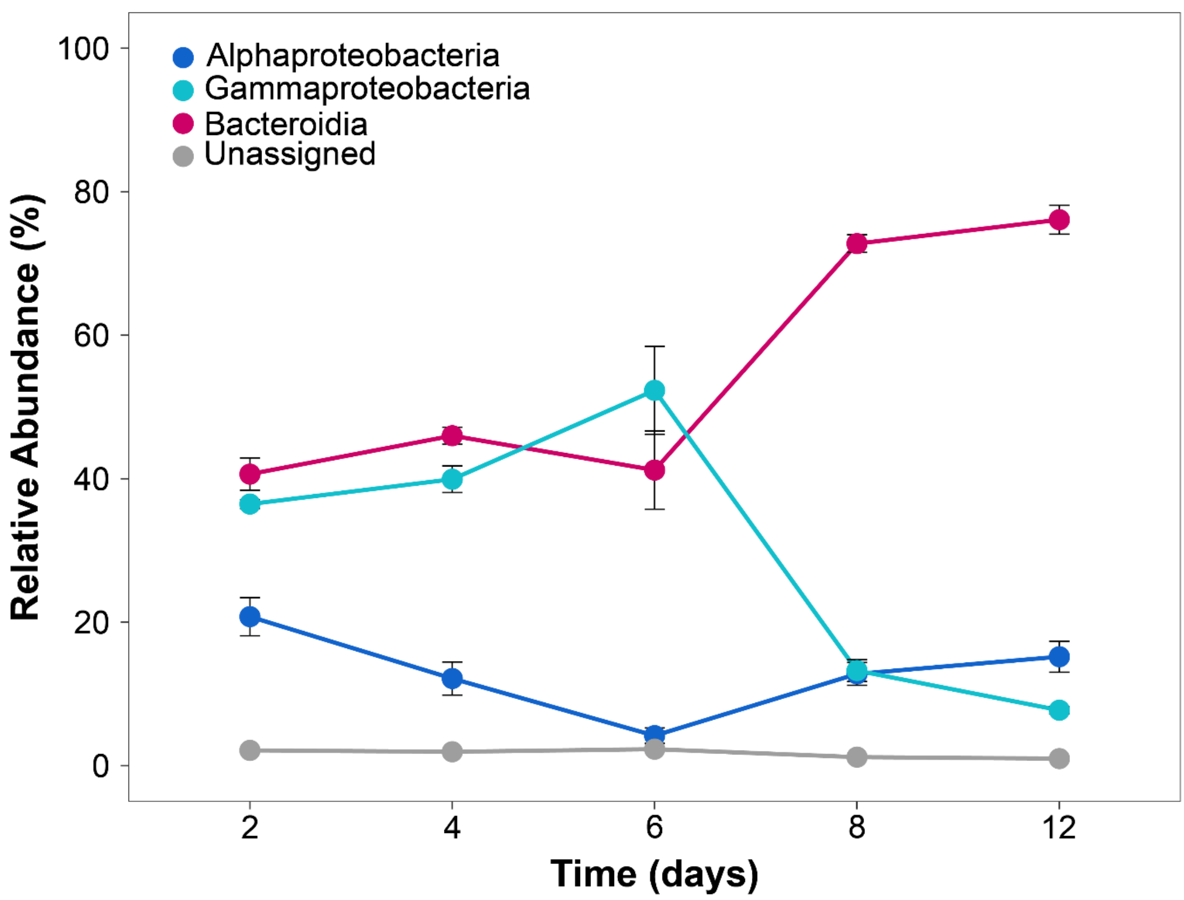

Figure S2: **Bacterial relative abundance through time in *Actinocyclus* sp. associated bacterial assemblages (Class level)**. Error bars represent the standard error of the mean (n=4).

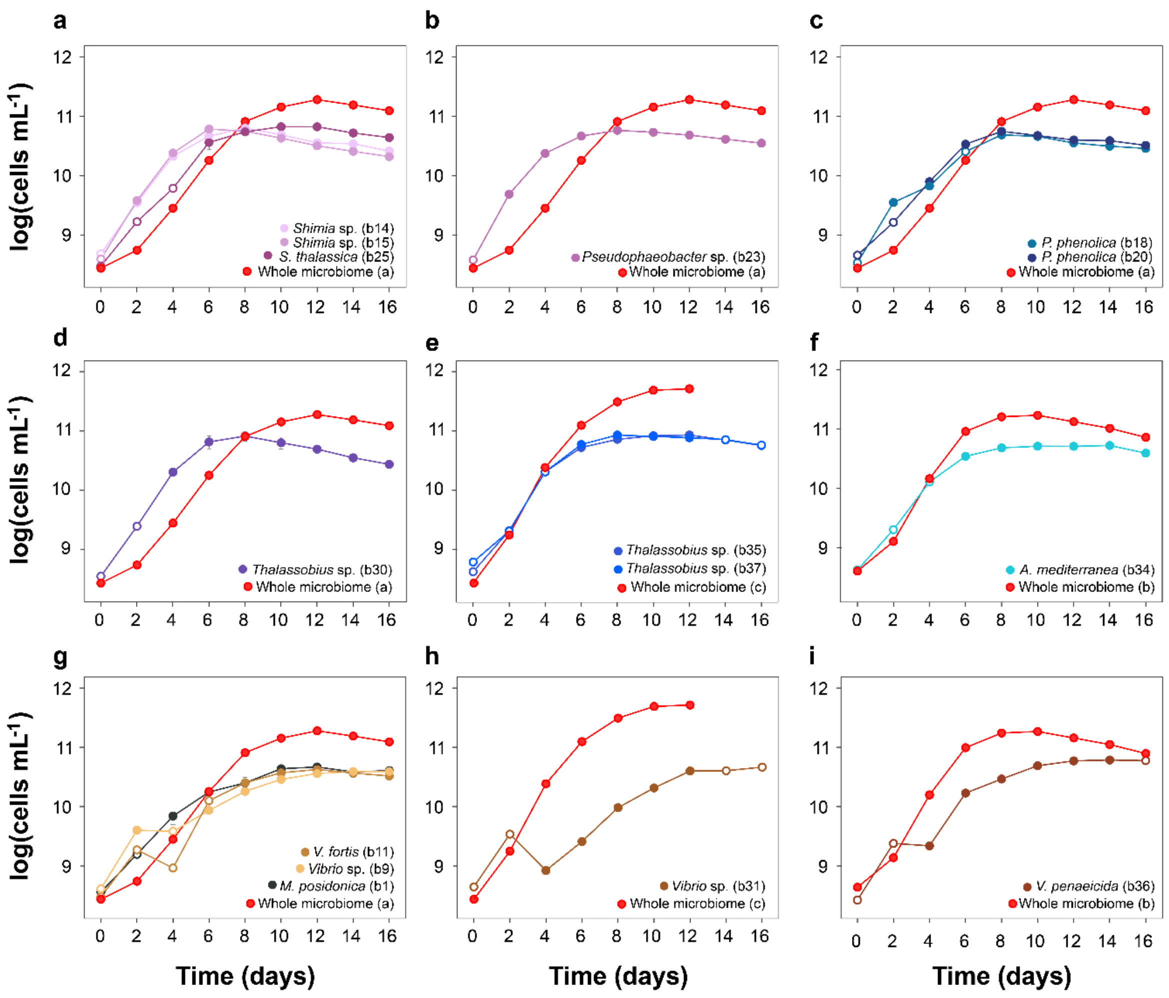

Figure S3: *Actinocyclus* sp. co-culture growth curves displaying log-transformed algae cells mL^{-1} with whole microbiome controls. a-d and g: co-cultures with the whole microbiome control (a)**; e, h:** co-cultures with the whole microbiome control (c)**; f, i:** co-cultures with the whole microbiome control (b). Filled circles refer to time points that were significantly different from the whole microbiome controls (Simple Main Effect test, $p < 0.05$, Table S16), while empty circles were not statistically different. Error bars represent the standard error of the mean (n=4) and numbers in parenthesis in the legend correspond to bacteria strain codes. Raw data are available in Table S10.

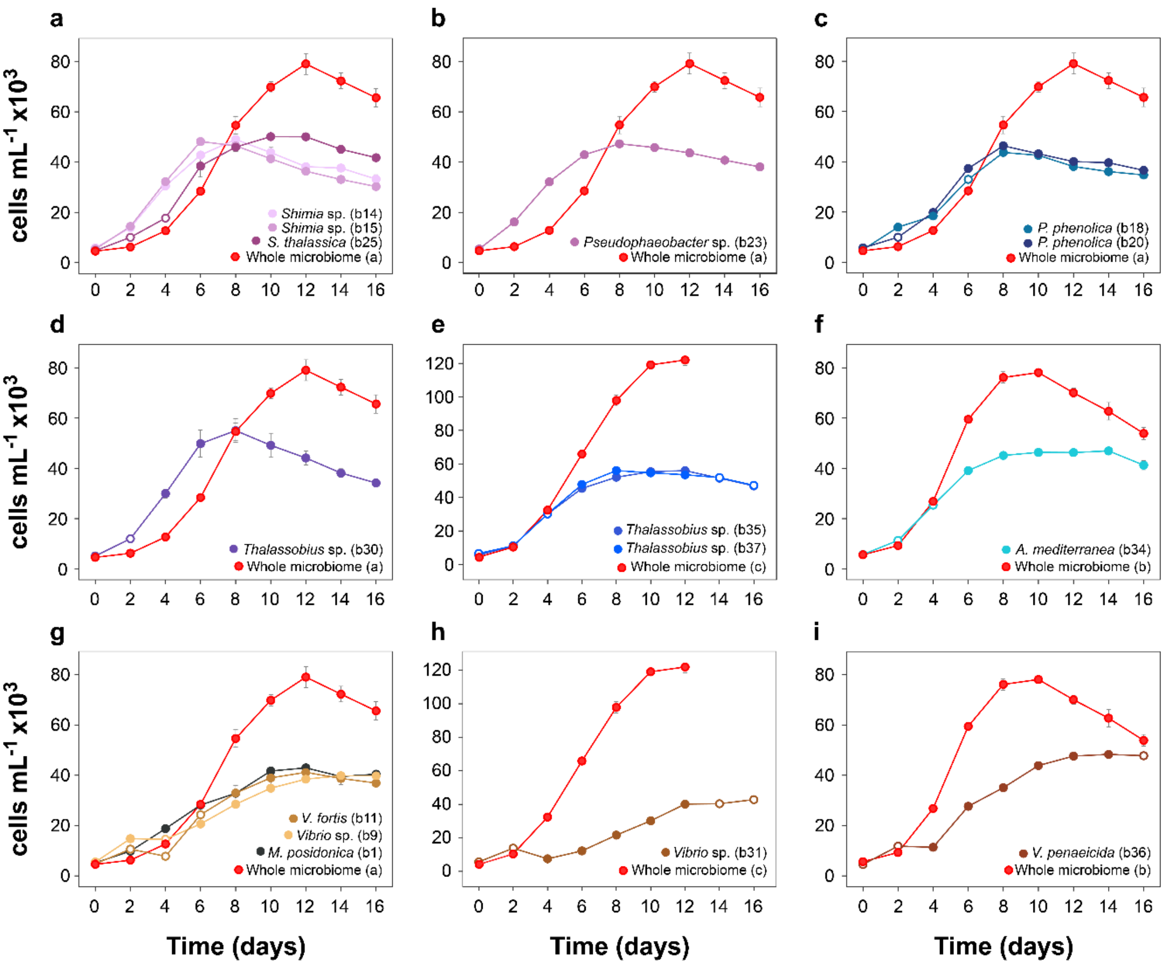

Figure S4: *Actinocyclus* sp. co-culture growth curves (±SEM) displaying algae cells mL^{-1} with whole microbiome controls. **a-d and g:** co-cultures with the whole microbiome control (a)**; e, h:** co-cultures with the whole microbiome control (c)**; f, i:** co-cultures with the whole microbiome control (b). Filled circles refer to time points significantly different from the whole microbiome controls (Simple Main Effect test, $p < 0.05$, Table S16), while empty circles were not statistically different. Error bars represent the standard error of the mean (n=4) and numbers in parenthesis in the legend correspond to bacteria strain codes. Red lines correspond to the whole microbiome control. Raw data are available in Table S10.

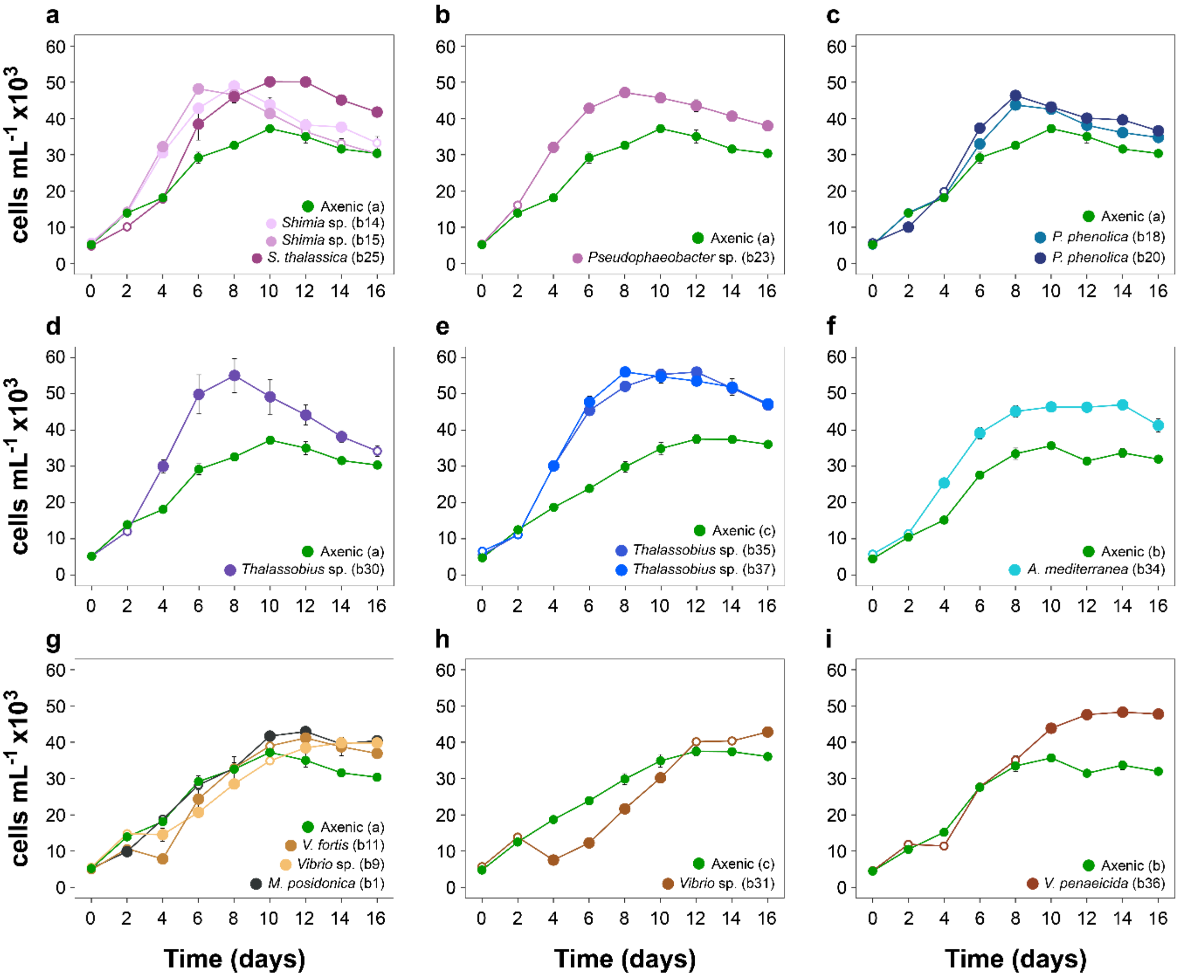

Figure S5: *Actinocyclus* sp. co-culture growth curves (±SEM) displaying algae cells mL^{-1} with axenic controls. a-d and g: co-cultures with the axenic control (a)**; e, h:** co-cultures with the axenic control (c)**; f, i:** co-cultures with the axenic control (b). Filled circles refer to time points significantly different from the axenic controls (Simple Main Effect test, $p < 0.05$, Table S4), while empty circles were not statistically different. Error bars represent the standard error of the mean (n=4) and numbers in parenthesis in the legend correspond to bacteria strain codes. Green lines correspond to the axenic control. Raw data are available in Table S10.

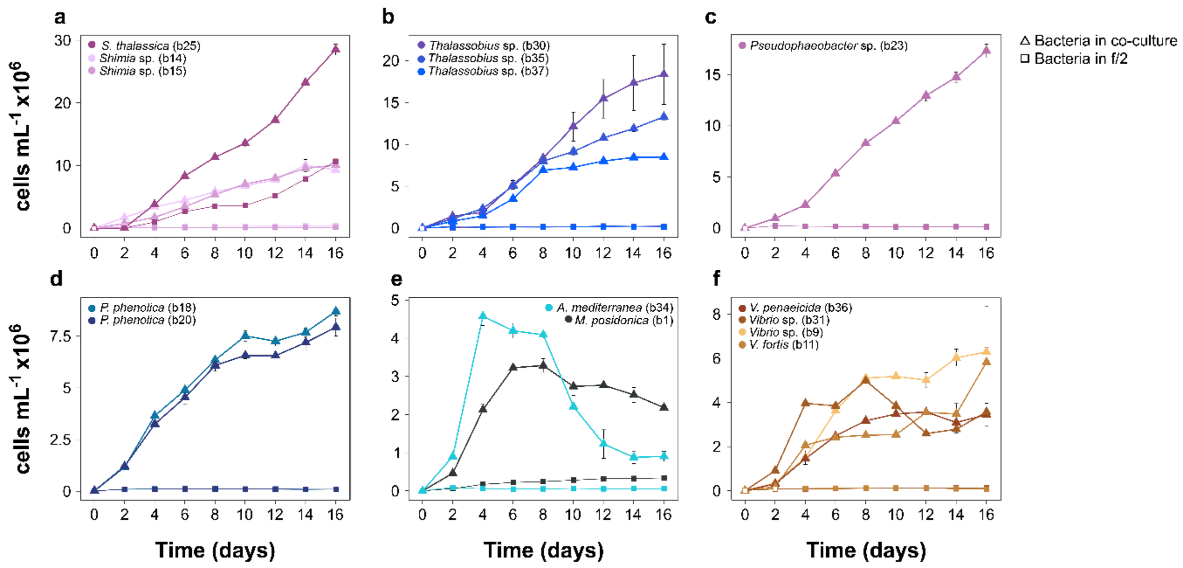

Figure S6: ***Actinocyclus* sp. co-culture growth curves displaying bacteria cells mL^{-1} against the bacteria in f/2 media controls**. **a-c**: Rhodobacteraceae co-cultures with their respective bacterial control in f/2; **d**: Pseudoalteromonadaceae co-cultures with their respective bacterial control in f/2; **e**: Alteromonadaceae and Oceanospirillaceae co-cultures with their respective bacterial control in f/2; f: Vibrionaceae co-cultures with their respective bacterial control in f/2. Errors bars represent the standard error of the mean (n=4). Bacteria control for *Vibrio penaeicida* (b36) is not presented due to contamination. Simple Main Effect Test ($p < 0.05$) between bacteria in co-cultures and controls in f/2 is available in Table S17. Note: flow cytometry does not take into account bacterial cells attached to the diatoms.

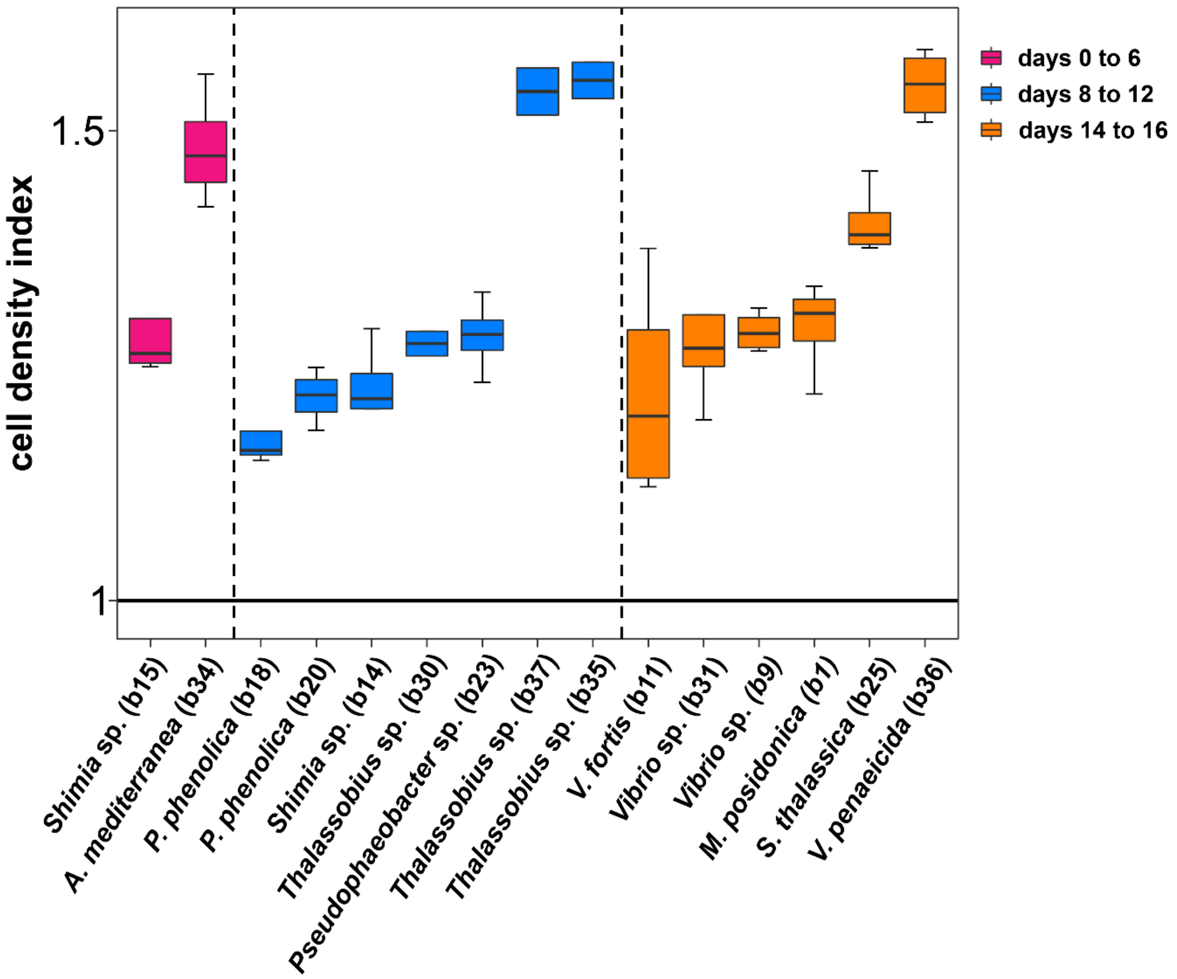

Figure S7: **Maximum cell density index of each bacterial isolate.** The time-period when the highest enhancement effect is displayed for each of the 15 bacterial isolates.

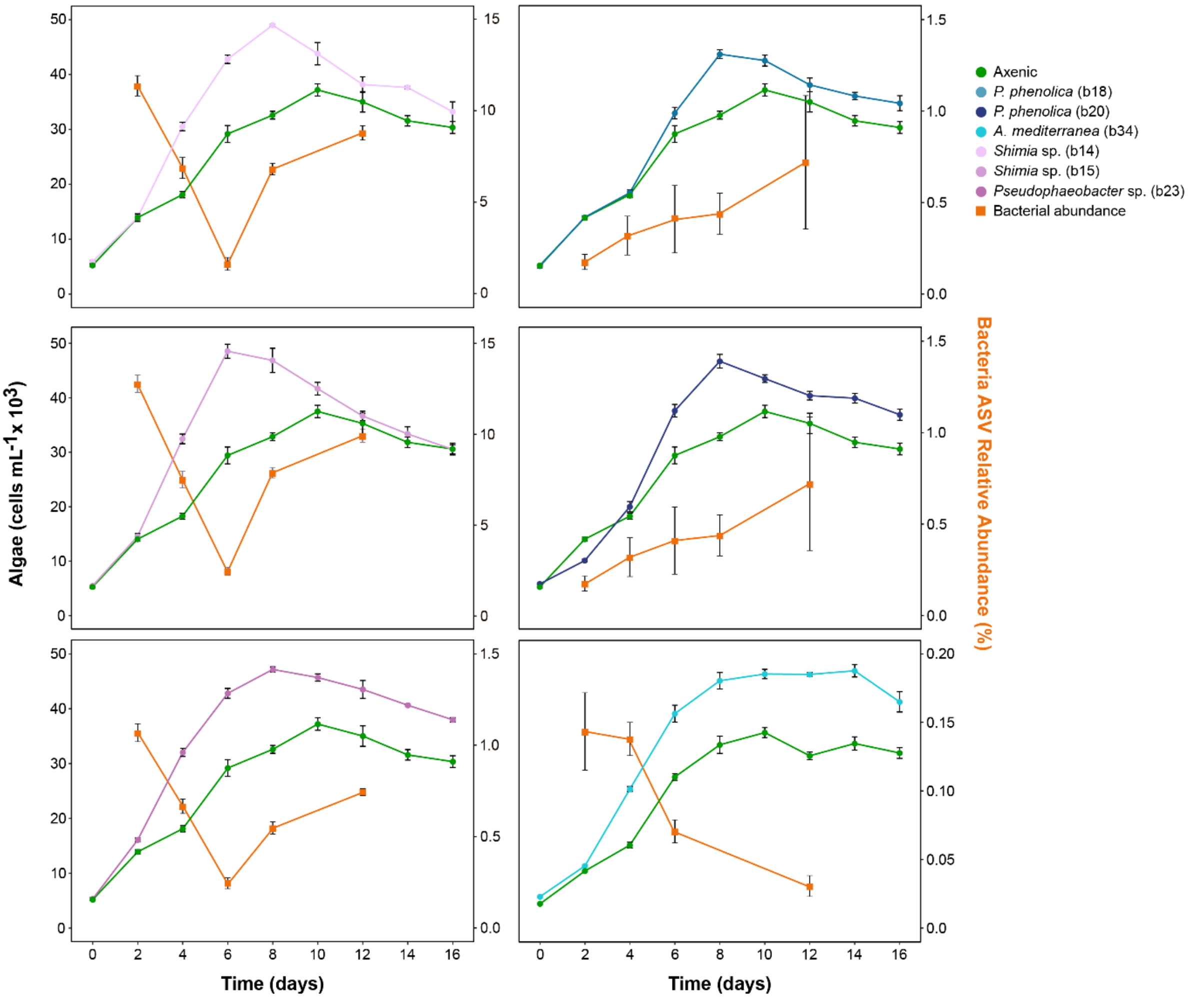

Figure S8: **Bacterial isolates ASVs within *Actinocyclus* sp. associated bacterial assemblages**. The relative abundance of exact bacterial isolates match (100% identity) within the microbiome (right y-axis) compared to the growth enhancement effect of those isolates grown in co-culture with the algae (in cells mL^{-1}, left y-axis). Error bars represent the standard error of the mean (n=4). The coloured line corresponds to the diatom concentration in co-culture with the different isolates, green lines correspond to the axenic controls and the orange lines correspond to the relative abundance of the ASVs within the microbiome of *Actinocyclus* that had a 100% identity match with the isolates used in co-culture.
